# Micelle and Nanotape Formation of Benzene Tricarboxamide Analogues with Selective Cancer Cell Cytotoxicity

**DOI:** 10.1021/acsomega.2c05940

**Published:** 2022-12-07

**Authors:** Nada Aljuaid, Jani Seitsonen, Janne Ruokolainen, Francesca Greco, Ian W. Hamley

**Affiliations:** † School of Chemistry, Pharmacy and Food Biosciences, University of Reading, Whiteknights, Reading RG6 6AD, U.K.; ‡ Nanomicroscopy Center, Aalto University, Puumiehenkuja 2, FIN-02150 Espoo, Finland

## Abstract

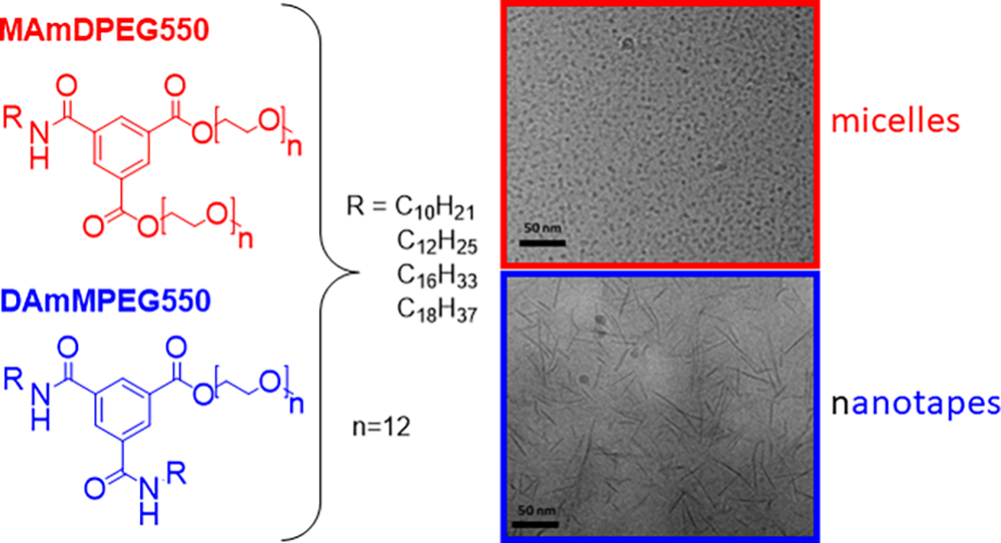

Analogues of benzene-1,3,5-tricarboxamide bearing combinations of different alkyl chains (dodecyl to octadecyl) and ester-linked PEG (polyethylene glycol) chains are shown to self-assemble into either micelles or nanotapes in aqueous solution, depending on the architecture (number of alkyl vs PEG chains). The cytotoxicity to cells is selectively greater for breast cancer cells than fibroblast controls in a dose-dependent manner. The compounds show strong stability, retaining their self-assembled structures at low pH (relevant to acidic tumor conditions) and in buffer and cell culture media.

## Introduction

Supramolecular polymerization via noncovalent interactions is emerging as a powerful method to create novel molecular assemblies with unique functions.^1−4^ Supramolecular polymerization often leads to one-dimensional self-assembly into extended fibril structures, although the mode of self-assembly can be tuned to generate other nanostructures, depending on molecular functionality. The self-assembly can be driven by a range of intermolecular interactions including hydrogen bonding, π-stacking, metal–ligand interactions, hydrophobic or solvophobic interactions etc.

Molecules based on benzene-1,3,5-tricarboxamide (BTA) have been shown to have many interesting properties,^2,3,5−8^ since they are model supramolecular polymers with a rigid π-stacking benzene motif surrounded by three hydrogen-bonding amide groups forming trifunctionalized self-assembling molecules. BTA derivatives have potential applications such as organo- and metallocatalysis^9−11^ and the development of biomaterials^12^ and stimuli-responsive materials.^13^ In another interesting recent example, BTA has been used as a scaffold to prepare trifunctional di- and tripeptide derivatives containing diphenylalanine as a common motif that favors β-sheet formation.^14^ These were found to self-assemble into one-dimensional twisted nanofibers. BTA derivatives have also attracted attention as model systems to study supramolecular polymerization.^13,15^

BTA derivatives with suitable hydrophilic substituents such as PEG, peptides, dendritic oligoglycerol, saccharides, or ureas can self-assemble in aqueous solution.^16−24^ We recently showed that BTA derivatives bearing combinations of lipid and PEG chains on the three arms self-assemble into different types of nanostructures depending on the balance of the numbers of alkyl and PEG chains. Specifically, spherical micelles were observed for derivatives with one alkyl and two PEG chains, but nanotapes and nanoribbons were revealed by SAXS and cryo-TEM for the series with two alkyl chains and one PEG chain.^25^

**Scheme 1:**
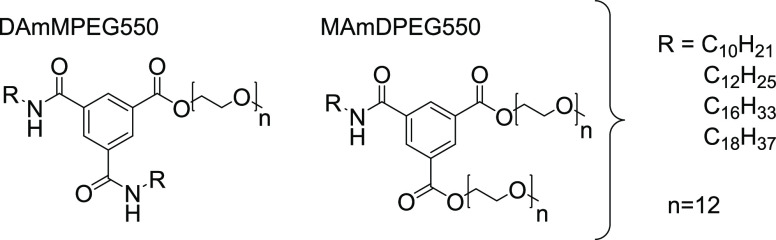
Schematic of the Scaffold of the BTA Analogues Studied

Here, we investigate the self-assembly in aqueous solution of new BTA analogues bearing combinations of one or two alkyl chains or ethylene glycol, EG (polyethylene glycol, PEG550, i.e., with approximately 12 EG repeats) as shown in Scheme 1. In contrast to our previous report, the EG chains are attached via ester rather than amide linkages. This confers responsiveness to esterases, which are overexpressed in tumor growth.^26−28^ The ester linkage also provides responsiveness to pH since esters are degraded under basic pH conditions, and last we wondered whether differences in conformation would influence the self-assembly behavior. All these aspects are examined here. We examined the cytotoxicity of all the synthesized compounds toward a model breast cancer cell line compared to fibroblast cell controls. Since tumors have an acidic microenvironment, we also investigated the stability of the observed self-assembled nanostructures against variation of pH. Sample names have the form DA*m*MPEG550 (*m* = number of carbon atoms in alkyl chain) for the compounds with two alkyl chains and a single PEG chain and MA*m*DPEG550 for compounds with one alkyl chain and two PEG chains. All samples were found to be soluble at pH 2, 7, and 12.

## Results and Discussion

The molecules were synthesized as described previously,^25^ following methods developed by Meijer, Palmans, and co-workers.^29^ A detailed scheme of the synthesis methods and extensive characterization data are provided in the Supporting Information (SI Schemes S1 and S2 and SI Figures S1–S19).

**Figure 1:**
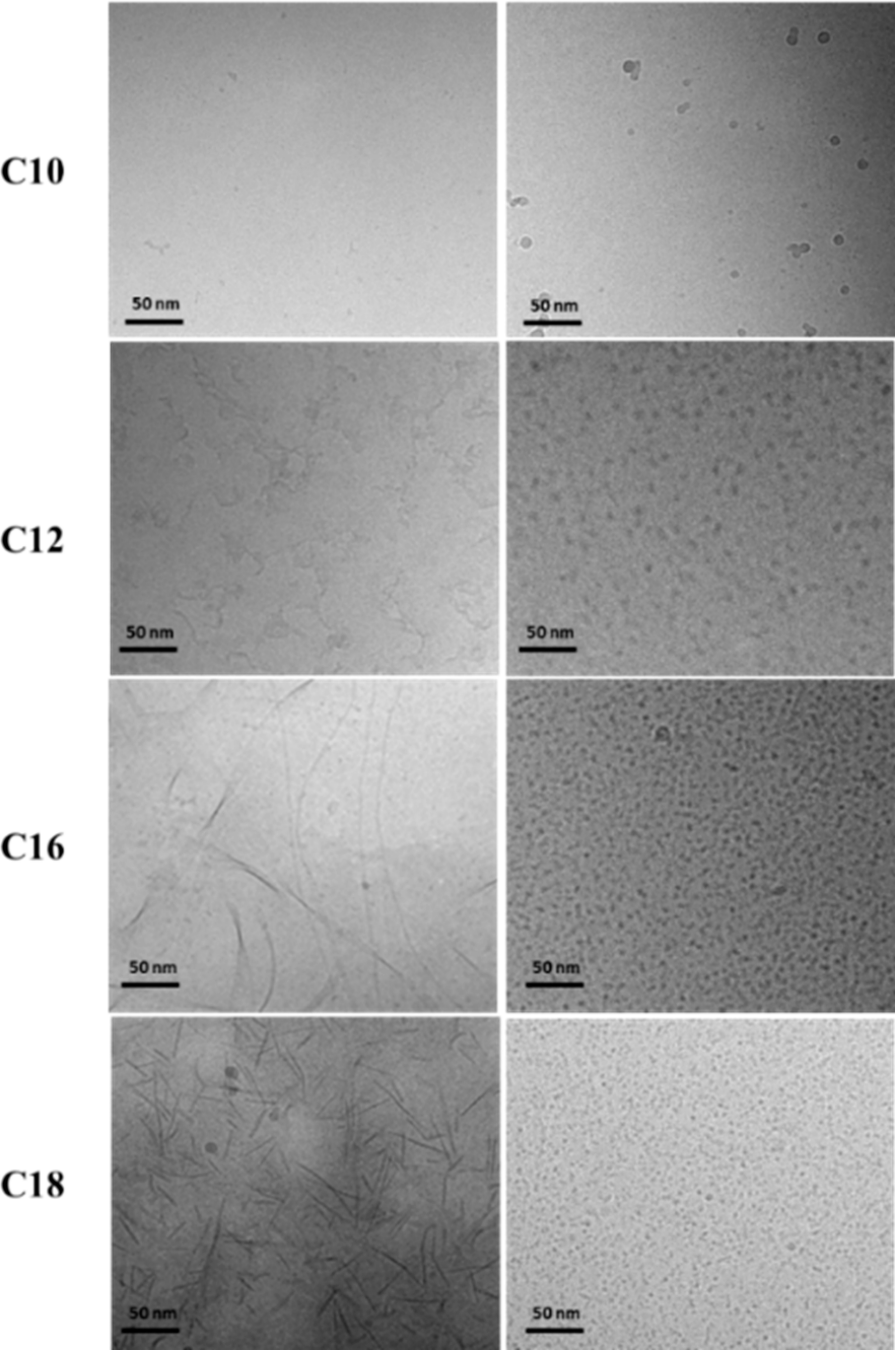
Cryo-TEM images for (left) DA*m*MPEG550 series and (right) the MA*m*DPEG550 series (all 1 wt % aqueous solutions).

Cryogenic transmission electron microscopy (cryo-TEM) was used to image nanostructures in aqueous solutions, and images are shown in Figure 1. The images reveal that the DA*m*MPEG550 samples form nonmicellar structures; in particular, twisted nanotapes are especially evident for samples with *m* = 16 and 18, and vesicles are occasionally seen for *m* = 12 (the *m* = 10 sample shows little evidence of self-assembled nanostructures). In complete contrast, the MA*m*DPEG550 samples form regular micelle structures with a defined diameter (ca. 6–8 nm) which are observed extensively over the cryo-TEM grid (Figure 1).

**Figure 2:**
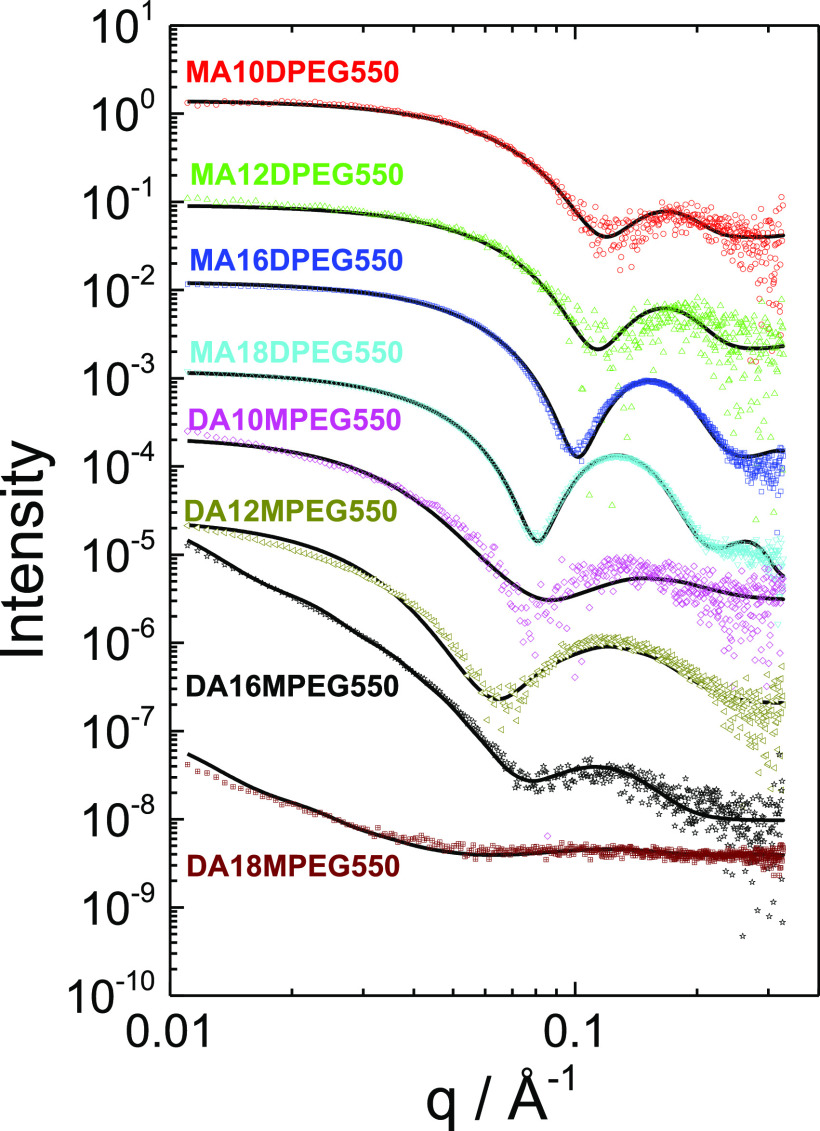
Measured SAXS data (open symbols) and fitted form factors (models and fitted parameters listed in SI Table S1). Data has been scaled to enable visualization, and only every 5th measured data point is shown.

Cryo-TEM was complemented with *in situ* small-angle X-ray scattering (SAXS) which provides detail on molecular form factors from which the shape and dimensions of nanostructures formed in aqueous solution can be determined.^30^ SAXS data measured at pH 7 is presented in Figure 2 which includes the measured intensity profiles along with form factor fits. The data falls into two classes consistent with the formation of distinct nanostructures for the two series of BTA analogues. Spherical micelles for the MA*m*DPEG550 series lead to intensity profiles showing a flat plateau at low wavenumber *q* and a well-defined form factor maximum at higher *q*. The latter feature arises from the local structure of a nanoscale assembly. In contrast, the intensity profiles for the DA*m*MPEG550 series show a finite slope at low *q* and a notably broad form factor maximum at high *q*. The form factor data were fitted^31^ using models for nanotapes (modeled with a bilayer electron density profile^32,33^) for the DA*m*MPEG500 series and spherical core–shell micelles^30^ for the MA*m*DPEG500 series, consistent with the morphologies from cryo-TEM shown in Figure 1.

The parameters from the fits at pH 7 are listed in SI Table S1. For the MA*m*DPEG550 samples, a clear trend of increasing core and outer radii with increasing alkyl chain length *m* can be seen. The radii are reasonable in view of the estimated molecular length; for example, for MA16DPEG550, the extended alkyl chain length is approximately 18 Å and the PEG radius of gyration (*R*_g_) is about 8 Å. The obtained inner core radii values listed in SI Table S1 are lower than the extended alkyl chain lengths, which may indicate folding; however, in addition the boundaries between core and shell will be diffuse and it also has to be considered that the BTA residues must presumably reside at the outer part of the core. The shell thickness (relatively constant, in the range 25–31 Å) is significantly larger than the PEG radius of gyration, indicating significant chain stretching. Another trend evident from the parameters for the MAmDEPG500 samples shows an increase in contrast (electron density) between the core and shell with increasing *m*; i.e., the longer alkyl chains are more tightly packed. For the DA*m*MPEG550 samples, the model form factor is one developed for lipid bilayers^32^ and represents the electron density profile across the bilayer as a sum of three Gaussian functions, one representing the hydrocarbon center of the bilayer (with a negative electron density relative to the solvent) and the other two (positive electron density contrast) Gaussians symmetrically representing the outer surfaces. The fit parameters indicate that the bilayer thickness increases with *m*. For *m* = 10, 12 the chains in the bilayer will be interdigitated, since the layer thicknesses are less than twice the estimated extended length of the alkyl chains; for example, for C_12_ chains, the chain length is approximately 12 Å whereas the layer thickness *t* = 27.1 Å according to the SAXS form factor fitting for DA10MPEG550 (SI Table S1) and the PEG chain outer layer thickness (8 Å) also has to be considered. However, the interdigitation is relaxed for larger *m*; for example, the layer thickness for the *m* = 18 sample is *t* = 45 Å. Also notable in the parameters for the DA*m*MPEG550 series is the increase in lateral dimensions of the bilayer structures with increasing *m* (also consistent with the low *q* slope of the form factors in Figure 2). This is in agreement with the appearance of longer nanotapes in the cryo-TEM images for the samples with *m* = 16, 18 (Figure 1). The difference in the slope of the intensity at low *q* for MA16DPEG550 and MA18DPEG550 may reflect interparticle aggregation (structure factor) effects.

The observation of spherical micelles for the MA*m*DPEG550 series and of nanotapes for the DA*m*MPEG550 series is consistent with our previous report^25^ for related BTA derivatives with different PEG chain lengths attached through amide linkers. Therefore, PEG chain length and the attachment through ester rather than amide linkages does not affect the self-assembly behavior. As with our prior report, the nanostructure formation can be rationalized on the basis of packing of the alkyl and PEG chains. Molecules bearing two PEG chains form micelles with a hydrated PEG shell and an alkyl chain as core, whereas the DA*m*MPEG550 BTA analogues form nanotapes based on bilayers with two interdigitated alkyl chains forming the hydrophobic sublayer, PEG forming the other hydrated layer.

**Figure 3:**
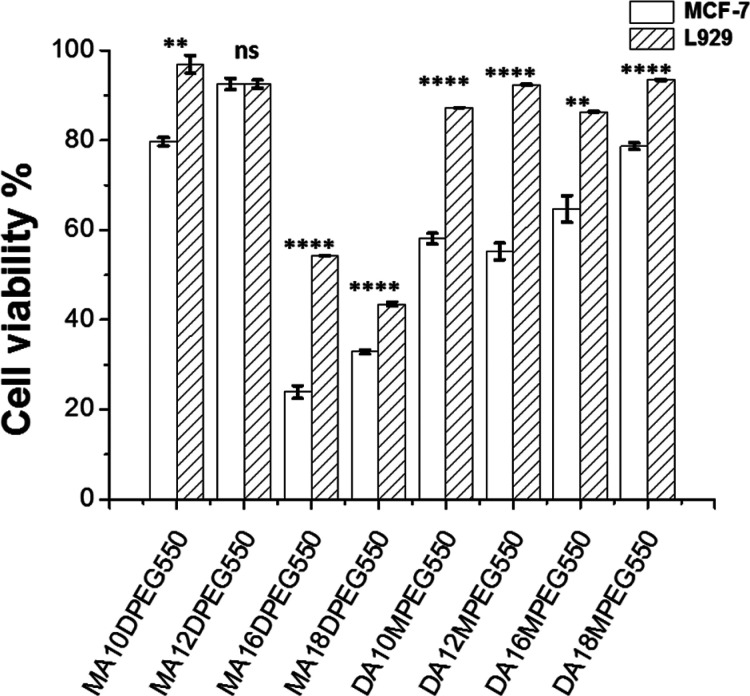
Cell viabilities from MTT [3-(4,5-dimethylthiazol-2-yl)-2,5-diphenyltetrazolium bromide] assays for L929 fibroblast and MCF-7 breast cancer cell lines for the compounds indicated at 10 μM concentration. Student’s *t*-test significance values *p* < 0.05 *, *p* < 0.01 **, *p* < 0.001 ***, and *p* < 0.0001 ****.

Although unmodified PEG itself is considered relatively benign to cells,^34,35^ and indeed is incorporated in certain therapeutics to provide enhanced stability and longer circulation in vivo, PEG derivatives can show cytotoxicity.^36^ The activity of the BTA analogues in terms of selective cytotoxicity against cancer cells was investigated using assays comparing the viabilities of L929 fibroblasts and MCF-7 breast cancer cells. Unexpected selective cytotoxic activity against cancer cells was found (Figure 3). However, for most compounds there was no significant difference between the viability for the two cell types at 1 μM due to low cytotoxicity (SI Figure S20) but significant differences emerged at a 10 μM dose (Figure 3) and also 100 μM (SI Figure S20). In general, the largest cell killing activity is observed for the MA*m*DPEG550 series with longer (C_16_ and C_18_) alkyl chains. The selectivity, however, depends on the composition of the BTA analogues. For the MA series, the longer chains (C_16_ and C_18_) are more cytotoxic, while for the DA series the trend appears to be opposite; i.e., they become more cytotoxic for the two shorter alkyl chain derivatives, again with selectivity for MCF-7 breast cancer cells.

In previous work, fibril-forming BTA analogues functionalized in all three arms with oligoethylene glycol (OEG176, i.e., 4 EG repeats) and/or mannose-terminated arms were found to have minimal cytotoxicity toward several cancer cell lines.^12^ The significant and selective cytotoxicity reported here results from the functionalization of BTA with both PEG and alkyl chains, especially for the monoalkyl derivatives. This leads to the formation of PEG-coated micelles which have greater cytotoxicity than BTA-based fibrils reported previously^12^ or BTA-nanotapes (observed here and in our previous report^25^) and which show selectivity to cancer cells. We suggest that this may be related to the distinct lipid composition of the membranes of cancer cells compared to normal cells.^37−39^ This in turn may influence the interaction with aggregates such as PEGylated micelles. It is interesting to note the higher cytotoxicity of the micelle-forming derivatives (with longer alkyl chains) which points to a role of the shape of the nanostructure on cytotoxicity, a feature already identified for inorganic nanoparticles, for example.^40^ The mechanisms by which the nanoassemblies interact with cell membranes is an interesting topic for future investigation but may involve micelle solubilization of the membrane and/or micelle disassembly at the cell wall. Compared to previous work using amide-linked BTA derivatives,^12^ it should be noted that the compounds in the present study contain ester linkages which may also influence cytotoxicity due for example to cell-expressed esterases.

The environment of a tumor is generally characterized by reduced pH;^41−43^ therefore, we examined the stability of our BTA analogues under acidic conditions. The nanostructures were conserved at pH 2 as confirmed by cryo-TEM images (shown for selected samples in SI Figure S21) which show nanotapes for the DA*m*MPEG550 series and micelles for the MA*m*DPEG550 series. This is further supported by SAXS data (for all samples) shown in SI Figure S22 which shows the same features as the data in Figure 2 for the samples at pH 7. In fact, the data can be fitted using many of the same fit parameters (in particular the layer thickness, *t*) as evident from comparison of SI Table S2 and SI Table S1. To cover basic conditions, measurements were also performed at pH 10. Under these conditions, the ester bonds are hydrolyzed, leading to degradation of the compounds as confirmed by ESI-MS which contains large peaks from the PEG chains. Representative data for DA16MPEG550 is shown in SI Figure S23 which also shows mass spectra at pH 2 compared to native pH 7, confirming the absence of degradation under these conditions. SAXS data also obtained for the samples under basic pH conditions (SI Figure S24) shows that the nanostructures formed at neutral and acidic pH are disrupted since the characteristic features of micelles or nanotapes are absent and/or only residual scattering signal was observed. The stability of the self-assembled nanostructures to changes in the aqueous medium were also examined. Cryo-TEM imaging was performed for samples in PBS and the media used for the cell viability studies (i.e., DMEM and RPMI-1640). The images shown in SI Figures S25–S17 confirm the presence of self-assembled structures in all of these solutions, and in addition, the distinct nanostructures for the DA*m*MPEG550 and DA*m*MPEG550 series observed in water (Figure 1) are largely preserved. Thus, our BTA analogues form self-assemblies that are highly stable against changes in pH as well as aqueous medium.

## Conclusions

In summary, we have shown that BTA analogues with dialkyl and mono-PEG functionalization self-assemble into nanotapes in aqueous solution, whereas monoalkyl and di-PEG substituents form micelles. The distinct nanostructures are formed primarily due to packing effects of the alkyl chains and PEG, but this may also also be influenced by hydrogen bond formation.

We also found unanticipated selective cytotoxicity toward cancer cells compared to fibroblasts, particularly notable for the longer monoalkyl chain compounds. The shorter dialkyl chain derivatives have lower cytotoxicity, although the selectivity is enhanced. The nanostructures for both MA and DA series are stable under acidic conditions but are disrupted by ester bond hydrolysis at basic pH. In other words, the derivatives show novel base-responsive disassembly and degradation which may be useful in the development of pH-responsive materials. The self-assembled nanostructures are exceptionally stable in buffer and cell media. Our findings should stimulate further research to examine the interaction of BTA analogues with different types of cells and membranes and additionally highlights the exceptional potential of BTA analogues to produce nanostructures of different dimensionality and to create novel bioactive materials.

## Supporting Information Available

Experimental procedures including materials and methods, synthesis details, schemes, and characterization data (MS and NMR), UV and FTIR spectra, additional SAXS data and cryo-TEM images, and tables listing the parameters extracted from the fitting to the SAXS data (PDF)
