# Supplementary material for: Micelle and Nanotape Formation of Benzene Tricarboxamide Analogues with Selective Cancer Cell Cytotoxicity
Source: ACS Omega. 2022 Dec 7;7(50):46843–8. doi: 10.1021/acsomega.2c05940 (PMC9773333; doi:10.1021/acsomega.2c05940)
Supplement: Supplementary file 1 — ao2c05940_si_001.pdf [file ao2c05940_si_001.pdf]

Supporting Information

Micelle and Nanotape Formation of Benzene Tricarboxamide Analogues with Selective Cancer Cell Cytotoxicity

Nada Aljuaid,[a] Jani Seitsonen,[b] Janne Ruokolainen,[b] Francesca Greco[a] and Ian W. Hamley[a,*]

[a] School of Chemistry, Pharmacy and Food Biosciences, University of Reading, Whiteknights, Reading RG6 6AD, UK

[b] Nanomicroscopy Center , Aalto University, Puumiehenkuja 2, FIN-02150 Espoo, Finland

* Author for correspondence

Materials

Reagents and solvents were purchased from Sigma Aldrich and used without further purification. Tetrahydrofuran (THF) was distilled under a nitrogen atmosphere from sodium benzophenone. Chloroform was distilled under a nitrogen atmosphere from calcium chloride. Synthesis details and characterization data are provided below.

Methods

Thin layer chromatography (TLC) was performed on aluminium sheets coated with Merck silica gel 60 F_{24}. Spots were visualised under ultra-violet light (254 nm) with potassium permanganate as the visualising agent. Column chromatography was performed using Merck silica gel 60 (40–63 μm particle size) and a mobile phase as specified. Melting points were recorded using a Stuart SMP10 melting point apparatus. ^{1}H NMR and ^{13}C NMR spectra were recorded using either $CDCl_3$ or DMSO-d_6 as solvent on either a Bruker Nanobay 400 or Bruker DPX 400 operating at 400 MHz for ^{1}H NMR or at 100 MHz for ^{13}C NMR. Mass spectrometry analysis was carried out using a Thermo-Fisher Scientific Orbitrap XL LC-MS. Fourier-Transform Infrared (FT-IR) spectra were measured using a Perkin-Elmer Spectrum 100 FTIR spectrometer set up with an ATR attachment allowing rapid recording of FTIR spectra that were scanned 32 times over the range 900−4000 cm^{-1}.

Cryogenic-Transmission Electron Microscopy (Cryo-TEM). Imaging was carried out using a field emission cryo-electron microscope (JEOL JEM-3200FSC), operating at 200 kV. Images were taken in bright field mode and using zero loss energy filtering (omega type) with a slit width of 20 eV. Micrographs were recorded using a Gatan Ultrascan 4000 CCD camera. The specimen temperature was maintained at -187 °C during the imaging. Vitrified specimens were prepared using an automated FEI Vitrobot device using Quantifoil 3.5/1 holey carbon copper grids with a hole size of 3.5 μm. Just prior to use, grids were plasma cleaned using a Gatan Solarus 9500 plasma cleaner and then transferred into the environmental chamber of a FEI Vitrobot at room temperature and 100 % humidity. Thereafter 3 μl of sample solution was applied on the grid and it was blotted twice for 5 seconds and then vitrified in a 1/1 mixture of liquid ethane and propane at temperature of -180 °C. The grids with vitrified sample solution were maintained at liquid nitrogen temperature and then cryo-transferred to the microscope.

Small-Angle X-Ray Scattering (SAXS). Synchrotron SAXS experiments on solutions were performed using a BioSAXS robots on beamline B21 at Diamond Light Source (Harwell, UK).[1] Solutions were loaded into the 96 well plate of an EMBL BioSAXS robot, and then injected via an automated sample exchanger into a quartz capillary (1.8 mm internal diameter) in the X-ray beam. The quartz capillary was enclosed in a vacuum chamber, in order to avoid air scattering. B21 operated with a fixed camera length (3.7 m) and fixed wavelength ($\lambda = 0.95$ Å).[1] SAXS patterns were captured using a Pilatus 2M detector. Data processing (background subtraction, radial averaging) was performed using the dedicated beamline software ScÅtter.

Cytotoxicity. *In vitro* cell culture studies were performed using a human breast adenocarcinoma cell line (MCF-7) and mouse fibroblast cell line (L929). Both were purchased from the European Collection of Cell Cultures (ECACC). MCF-7 cells were cultured in RPMI-1640 with L-glutamine medium supplemented with 5% fetal bovine serum (FBS). L929 Cells were cultured in Dulbecco's modified eagle's medium (high glucose) supplemented with 2 mM of L-glutamine, 10% FPS, penicillin (100 units per mL) and streptomycin (0.1 mg mL^{-1}). Cells were maintained in a 5% CO_2 humidified atmosphere at 37 °C. Cytotoxicity was examined using the MTT (3-(4,5-dimethylthiazol-2-yl)-2,5-diphenyltetrazolium bromide) assay. PEG550 samples were dissolved in complete medium in different concentrations. Cells were seeded into a 96-well plate at a seeding density of 4×10^4 cells/mL for MCF-7 and 4×10^4 cells/mL for L929 cells and allowed to adhere for 24 h in 100 μL of complete medium. After 24 h, a total volume of 100 μL of the BTA analogues was added to give concentrations 1 μM, 10 μM and 100 μM. After 67 h of incubation, a 3-[4,5-dimethylthiazol-2-yl]-2,5-diphenyltetrazolium bromide (MTT) solution (5 mg/mL in phosphate-buffered saline (PBS)) was added (20 μL per well) and allowed to incubate for 5 h (total of 72 h of incubation). After this, the solution was removed from each well and replaced with 100 μL of dimethyl sulfoxide ($DMSO_4$) per well to dissolve the formazan crystals. Plates were incubated for 30 min and then analysed using a UV microplate reader ($\lambda = 560$ nm). Results are reported as a percentage cell viability compared with control (untreated values). All assays were performed in triplicate.

Synthesis Details:

Synthesis of DA10MPEG550, DA12MPEG550, DA16MPEG550 and DA18MPEG550

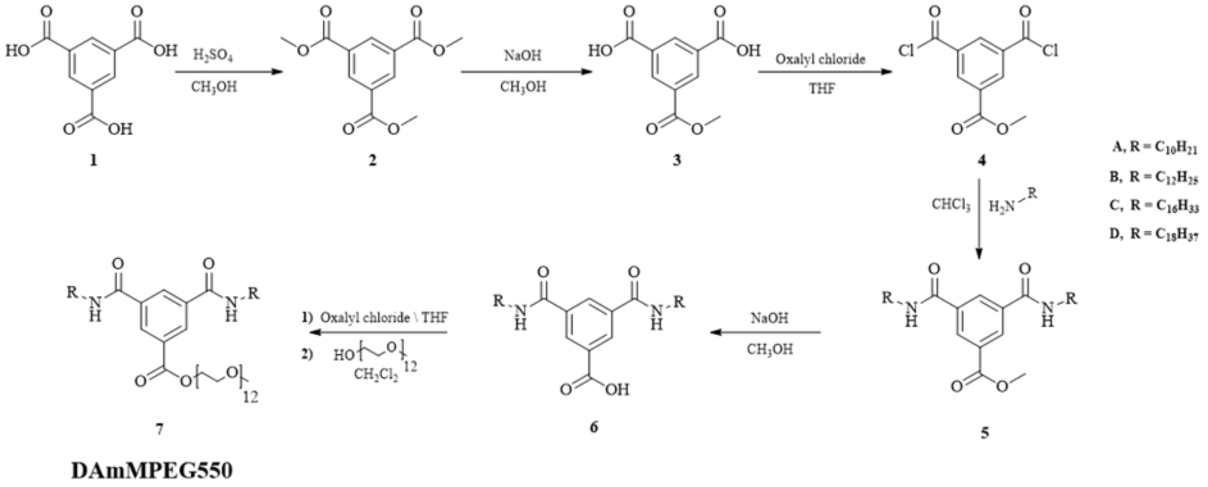

Scheme S1. Reaction scheme for DAmMPEG550 compounds

Trimethyl benzene-1,3,5-tricarboxylate (2). Trimesic acid **1** (8.00 g, 38.07 mmol) was dissolved in a mixture of methanol (150 mL) and conc. H_2SO_4 (2 mL, 38.07 mmol) and the mixture stirred under reflux for 24 hours at 85 ˚C. Subsequently, the solvent was removed under vacuum and the residue dissolved in chloroform (150 mL) and washed with saturated sodium bicarbonate (200 mL). The organic layer was collected and the solvent was removed to obtain the desired product as a white powder (8.84g, 92%); IR (ATR) ν/cm^{-1}: 1724.7 (C = O, ester), 1237.7 (C - O, ester), 1445.7 (C = C, Aromatic); ^{1}H NMR (400 MHz, $CDCl_3$) δ ppm: 8.86 (3H, s, Aromatic), 3.98 (9H, s, -OC*H*$_3$); ^{13}C NMR (100 MHz, $CDCl_3$) δ ppm: 165.41, 134.59, 131.20, 52.64; ESI-MS: *m/z* calculated for $[C_{12}H_{12}O_6 + H]^+$ = 253.22 Da Found = 253.07 Da.

Synthesis of 5-methoxycarbonyl-benzene-1,3-dicarboxylic acid (3). Trimethyl benzene-1,3,5-tricarboxylate **2** (5 g, 19 mmol) and 2.3 eq. NaOH (1.7 g, 44 mmol) were dissolved in MeOH (350 mL). The mixture was stirred for 24 hours under reflux at 85 °C, after which the mixture was allowed to cool down to room temperature. Thereafter, the mixture was concentrated to about 150 mL under vacuum, poured in 400 mL 1 M HCl in a separation funnel of 1 L and extracted with diethyl ether (3 x 300 mL). The organic layers were collected and the solvent was removed under vacuum. The crude product was recrystallized twice from ethyl acetate and obtained as a white solid (2.9 g, 61%); IR (ATR) ν/cm^{-1}: 3248 (O-H, carboxylic acid), 1716 (C = O ester), 1187 (C - O ester), 1436 (C = C, Aromatic) ; ^{1}H NMR (400 MHz, DMSO-d$_6$) δ ppm: 13.68 (1H, s, O*H*), 8.68 (3H, s, Aromatic), 3.95 (3H, s, O-C*H*$_3$); ^{13}C NMR(100 MHz, DMSO-d$_6$) δ ppm: 165.72 (O = *C* = OH), 164.85 (O = *C* - CH_3) 133.87 (C=*C*,

Ar), 133.34 (C=*C*-COH) 130.68 (C=*C*-COCH$_3$), 52.68 (*C*H$_3$); ESI-MS: *m/z* calculated for $[C_{10}H_7O_6+H]^+$ = 239.05 Da Found = 239.20 Da.

Synthesis of methyl-3,5-bis-chlorocarbonyl-benzoate (4). 5-methoxycarbonylbenzene-1,3-dicarboxylic acid (1.0g, 4.46 mmol) was dissolved in 30 mL of dry THF under a nitrogen atmosphere and a catalytic amount of DMF (~ 2 droplets). To this solution was added dropwise a solution of 2.5 eq. oxalyl chloride (0.95 mL, 11.15 mmol) in 10 mL of dry THF. The reaction was stirred for 90 min at room temperature (completion checked with ^{1}H-NMR), after which the THF was removed *in vacuo* and the excess of oxalyl chloride was removed by co-evaporation with toluene (3 x 50 mL). The product was obtained as a yellow oil (1.12g, 97%). IR (ATR) *ν*/cm^{-1}: 1701 (C = O acyl chloride), 1698 (C = O ester), 1238 (C - O ester), 737 (C - Cl acyl chloride); ^{13}C NMR (100 MHz, CDCl$_3$) δ ppm: ^{13}C NMR: δ =163.85 (O = *C* = Cl), 165.71 (O=*C*-CH$_3$), 133.86 (*C*=C-COCH$_3$, Aromatic), 133.36 (*C* = C-COCl), 132.07 (*C*-CCl), 129.83 (*C*-COCH$_3$), 52.70 (*C*H$_3$).

Synthesis of methyl-3,5-bis-n-Alkylaminocarbonyl-benzoate (5). A solution of 2 eq. Alkylamine and 3 eq. triethylamine was dissolved in dry CHCl$_3$ in ice bath under a nitrogen atmosphere. To this solution was added dropwise a solution of methyl-3,5-bis-chlorocarbonyl-benzoate **4** (1 eq.) in dry CHCl$_3$. The reaction was stirred for 24 hours at room temperature under nitrogen atmosphere. Then, the solvent was removed under vacuum and the resulting crude product was purified with a short silica-gel (CH$_2$Cl2: MeOH; 100:2) obtained a white product.

5A: (0.41g, 70 %). IR (ATR) *ν*/cm^{-1}: 2936 (C – H alkyl), 1726 (C = O ester), 1665 (C = O amide), 1392 (C-N amide), 1187 (C - O ester) ; ^{1}H NMR (400 MHz/CDCl$_3$) δ ppm: δ 8.52 (2H, d, J=1.7 Hz, Ar-*H*), 8.41 (1H, t, J= 1.8 Hz) 3.97 (3H, s, O-C*H$_3$*), 3.48 (4H, m, N-C*H$_2$*-),1.63 (4H, m), 1.32(28H, m), 0.87(6H, t, J= 6.8 Hz); ^{13}C NMR(100 MHz, CDCl$_3$) δ ppm: 165.5, 165.1, 133.1, 130.2, 52.8, 38.4, 31.8, 29.2, 26.7, 23.2, 14.3; ESI-MS: *m/z* calculated for $[C_{30}H_{50}N_2O_4+H]^+$ = 503.74 Da Found = 503.38 Da.

5B: (0.42g, 64 %). IR (ATR) *ν*/cm^{-1}: 2924 (C – H alkyl), 1723 (C = O ester), 1665 (C = O amide), 1376 (C-N amide), 1169 (C - O ester) ; ^{1}H NMR (400 MHz/CDCl$_3$) δ ppm: δ 8.52 (2H, d, J=1.7 Hz, Ar-*H*), 8.41 (1H, t, J= 1.8 Hz), 6.33 (2H, t, J= 5.9, N-H) 3.97 (3H, s, O-C*H$_3$*), 3.48 (4H, m, N-C*H$_2$*-),1.63 (4H, m), 1.32(36H, m), 0.87 (6H, t, J= 6.8 Hz); ^{13}C NMR(100 MHz, CDCl$_3$) δ ppm: 165.8, 165.5, 135.5, 131.1, 129.7, 52.7, 40.4, 31.9, 29.7, 29.6, 29.5, 29.4, 29.3, 27.1, 22.7, 14.1; ESI-MS: *m/z* calculated for $[C_{34}H_{58}N_2O_4+H]^+$ = 559.85 Da Found = 559.38 Da.

5C: (0.51 g, 66 %). IR (ATR) *ν*/cm^{-1}: 2921 (C – H alkyl), 1719 (C = O ester), 1662 (C = O amide), 1374 (C-N amide), 1165 (C - O ester) ; ^{1}H NMR (400 MHz/CDCl$_3$) δ ppm: δ 8.52 (2H, d, J=1.7 Hz, Ar-*H*), 8.41 (1H, t, J= 1.8 Hz), 6.33 (2H, t, J= 5.9, N-H) 3.97 (3H, s, O-C*H$_3$*), 3.48 (4H, m, N-C*H$_2$*-),1.63 (4H, m), 1.32(52H, m), 0.87 (6H, t, J= 6.8 Hz); ^{13}C NMR(100 MHz, CDCl$_3$) δ ppm: 165.8, 165.5, 135.5, 131.1, 129.7, 52.7, 40.4, 31.9, 29.7, 29.6, 29.5, 29.4, 29.3, 27.1, 22.7, 14.1; ESI-MS: *m/z* calculated for $[C_{42}H_{74}N_2O_4+H]^+$ = 671.06 Da Found = 671.57 Da.

5D: (0.75 g, 68 %). IR (ATR) *ν*/cm^{-1}: 2927 (C – H alkyl), 1721 (C = O ester), 1662 (C = O amide), 1372 (C-N amide), 1167 (C - O ester) ; ^{1}H NMR (400 MHz/CDCl$_3$) δ ppm: δ 8.52 (2H, d, J=1.7 Hz, Ar-*H*), 8.41 (1H, t, J= 1.8 Hz), 6.28 (2H, t, J= 5.9, N-H) 3.97 (3H, s, O-

CH_3), 3.48 (4H, m, N-CH_2-),1.63 (4H, m), 1.32(60 H, m), 0.87 (6H, t, J= 6.8 Hz); ^{13}C NMR(100 MHz, $CDCl_3$) δ ppm: 165.6, 165.3, 135.2, 131.1, 129.5, 52.4, 40.6, 31.9, 29.7, 29.6, 29.5, 29.4, 29.3, 27.0, 22.6, 14.1; ESI-MS: *m/z* calculated for $[C_{46}H_{82}N_2O_4+H]^+$ = 727.17 Da Found = 727.63Da.

Synthesis of 3,5-bis-n-Alkylcarbamoyl benzoic acid (6). 1 eq. methyl-3,5-bis-*n*-Alkylaminocarbonylbenzoate and 2 eq. of NaOH (4M) were dissolved in methanol. The solution was stirred and reflux at 85 ˚C for 24h after which the solution was cooled and poured into 300 mL H_2O that acidified with 1 M HCl. A white powder precipitated and was isolated by filtration. The product was dried under vacuum.

6A: (1.1, 67%). IR (ATR) *v*/cm^{-1}: 3358 (N-H amide), 2917 (O– H carboxylic acid), 1704 (C = O carboxylic acid), 1630 (C = O amide); ^{1}H NMR (400 MHz, DMSO-d_6) δ ppm: 8.76 (2H, t, J= 5.6 MHz), 8.51 (3H, s, Ar-*H*), 8.40 (1H, s, Ar-*H*), 3.27 (4H, m, NH-CH_2), 1.25 (28H, m, CH_2), 0.85 (6H, m, CH_3); ^{13}C NMR (100 MHz, DMSO-d_6) δ ppm: 166.4, 165.5, 165.3, 135.2, 130.4, 129.5, 31.8, 29.7, 29.6, 29.5, 29.4, 29.3, 27.1, 22.7, 14.1; ESI-MS: *m/z* calculated for $[C_{29}H_{48}N_2O_4+H]^+$ = 489.71 Da Found = 489.36 Da.

6B: (1.3, 66%). IR (ATR) *v*/cm^{-1}: 3291 (N-H amide), 2912 (O– H carboxylic acid), 1702 (C = O carboxylic acid), 1625 (C = O amide); ^{1}H NMR (400 MHz, DMSO-d_6) δ ppm: 8.79 (2H, t, J= 5.6 MHz), 8.55 (1H, s, Ar-*H*), 8.51 (2H, s, Ar-*H*), 3.27 (4H, m, NH-CH_2),1.53 (4H, m) 1.25 (36H, m, CH_2), 0.85 (6H, m, CH_3); ^{13}C NMR (100 MHz, DMSO-d_6) δ ppm: 166.4, 165.5, 165.3, 135.2, 130.4, 129.5, 31.8, 29.7, 29.6, 29.5, 29.4, 29.3, 27.1, 22.7, 14.1; ESI-MS: *m/z* calculated for $[C_{33}H_{56}N_2O_4+H]^+$ = 545.42 Da Found = 545.82 Da.

6C: (1.25 g, 62 %). IR (ATR) *v*/cm^{-1}: 2834 (O– H carboxylic acid), 1686 (C = O carboxylic acid), 1649 (C = O amide); ^{1}H NMR (400 MHz, DMSO-d_6) δ ppm: 8.55 (s, 2H, Ar-*H*), 8.40 (s, 1H, Ar-*H*), 7.16 (t, 2H, N-*H*), 3.47 (m, 4H, NH-C*H2*), 1.66- 1.28 (m, 36H, C*H2*), 0.89 (m, 6H, C*H3*). ^{13}C NMR (100 MHz, DMSO-d_6) δ ppm: 167.6, 165.8, 165.5, 135.5, 131.1, 129.5, 52.7, 31.8, 29.7, 29.6, 29.5, 29.4, 29.3, 27.1, 22.7, 14.1; ESI-MS: *m/z* calculated for $[C_{42}H_{74}N_2O_4+H]^+$ = 657.04 Da Found = 657.55 Da.

6D: (0.18, 65 %). IR (ATR) *v*/cm^{-1}: 3356(N-H amide), 2923 (O– H carboxylic acid), 1711 (C = O carboxylic acid), 1627 (C = O amide); ^{1}H NMR (400 MHz, DMSO-d_6) δ ppm: 8.76 (2H, t, J= 5.6 MHz), 8.51 (2H, t, Ar-*H*), 8.40 (1H, d, Ar-*H*), 3.27 (4H, m, NH-CH_2), 1.25 (28H, m, CH_2), 0.85 (6H, m, CH_3); ^{13}C NMR (100 MHz, DMSO-d_6) δ ppm: 166.3, 165.4, 165.3, 135.4, 130.4, 129.5, 31.8, 29.7, 29.6, 29.5, 29.4, 29.3, 27.1, 22.7, 14.1; ESI-MS: *m/z* calculated for $[C_{33}H_{80}N_2O_4+H]^+$ = 713.15 Da Found = 713.61 Da.

General Synthesis for of DAmMPEG550 (7). As a first step 3,5-bis-n-alkylcarbamoyl-benzoyl chloride was synthesised following the same procedure as described for 4, 1 eq. of 3,5-bis-n-Alkylcarbamoyl benzoic acid 6 and 1.5 eq. of oxalylchloride under nitrogen atmosphere yielding yellowish product. The resulted product was dissolved in dry THF and added slowly

to a solution of Poly (ethylene glycol) methyl ether (Mn 550) 1 eq. and triethylamine 2 eq. in dry THF in ice bath under a nitrogen atmosphere. The reaction was stirred overnight at room temperature under nitrogen atmosphere. Then, the solvent was removed under vacuum and the resulting crude product was purified by column chromatography.

DA10MPEG550. The synthesis of DA10MPEG550 was performed following the same procedure as described for **7,** was used Poly (ethylene glycol) methyl ether (Mn 550) (0.35 mL, 0.59 mmol) and triethylamine (0.15 mL, 1.48 mmol) in 15 mL dry THF in 0 ˚C under a nitrogen atmosphere. To this solution, a solution of 3,5-bis-n-decylaminocarbonyl-benzoyl chloride (0.25 g, 0.49 mmol) in 5 mL of dry THF. The reaction was stirred overnight at room temperature under nitrogen atmosphere. Then, the solvent was removed under vacuum and the resulted crude product was purified with silica-gel ($CHCl_3$: MeOH; 100:7) obtain a yellowish oil (0.2 g, 40 %). IR (ATR) *ν*/cm^{-1}: 3295 (N-H, amide), 2918 (C – H alkyl), 2848 (C – H PEG), 1726 (C = O ester), 1648 (C = O amide), 1250 (C-CO), 1093 (C - O ester); ^{1}H NMR (400 MHz/$CDCl_3$) δ ppm: δ 8.60 (2H, s), 8.46 (1H, s), 6.76 (2H, N-H), 4.53(2H, t, J=4.8 Hz), 3.85 (2H, t, J=4.8Hz), 3.61 (40H, m), 3.46 (4H, q, J= 6.8 Hz), 3.38 (3H, s), 1.65 (4H, m), 1.26 (28 H, m), 0.88 (6H, t, J= 6.4 Hz); ^{13}C NMR(100 MHz, $CDCl_3$) δ ppm: 165.6, 165.3, 135.5, 130.9, 130.7, 129.7, 71.9, 70.7, 70.6, 70.5, 70.4, 69.0, 64.6, 59.0, 40.4, 31.9, 29.6, 29.5, 29.4, 29.3, 27.0, 22.6, 14.1; ESI-MS: *m/z* calculated for $[C_{54}H_{98}N_2O_{16}]^+$ = 1030.69 Da Found = 1030.73 Da.

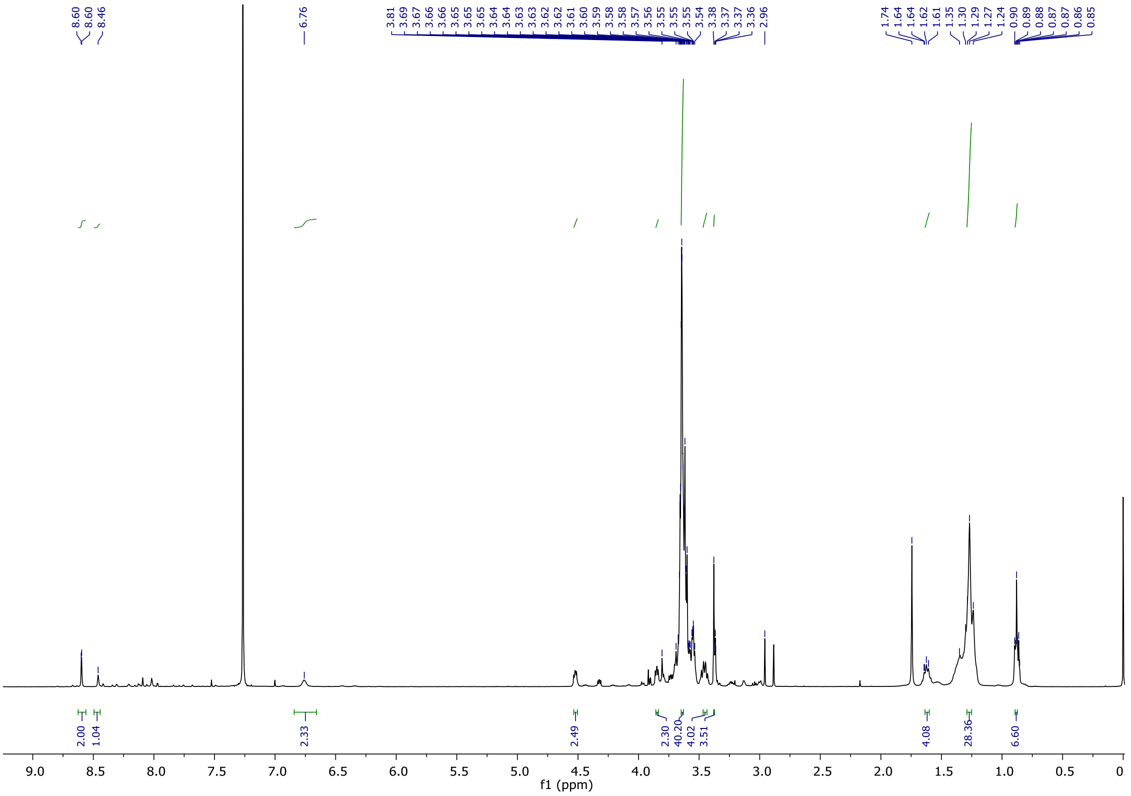

Figure S1: ^{1}H NMR spectrum of DA10MPEG550

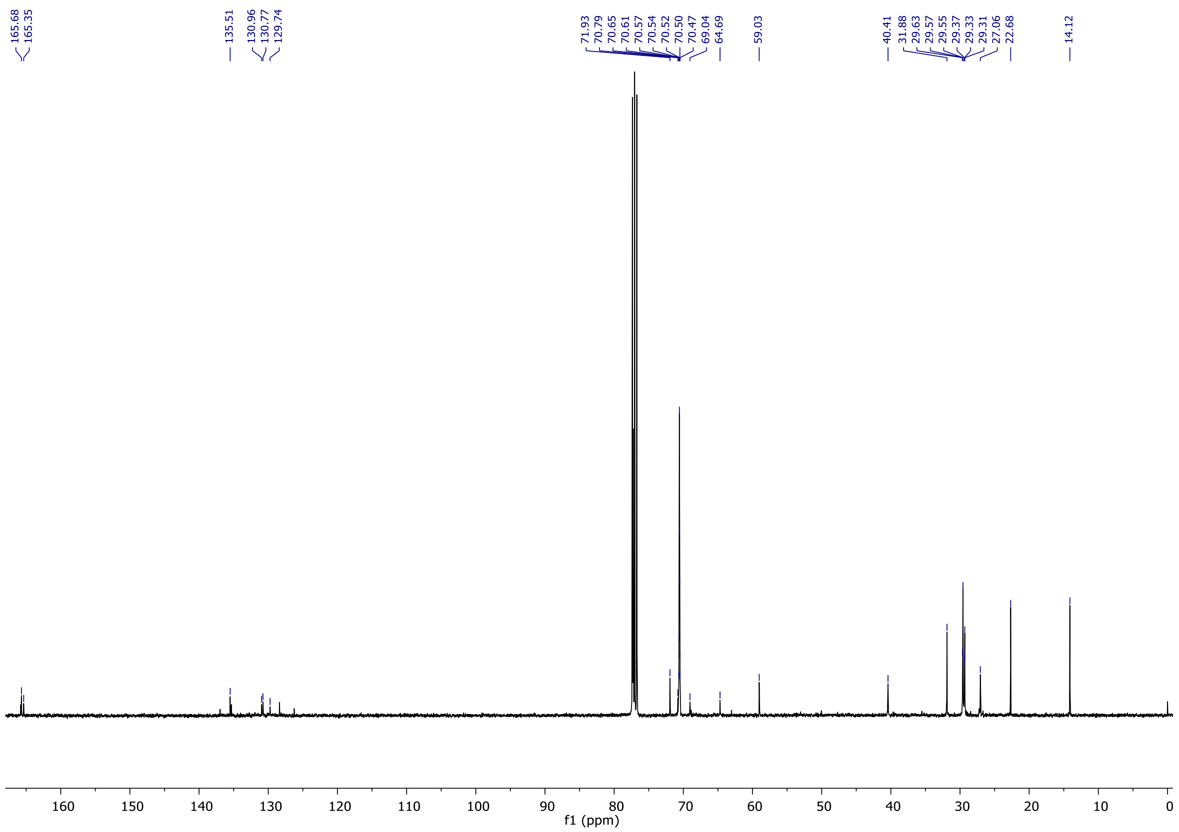

Figure S2: ^{13}C NMR spectrum of DA10MPEG550

DA12MPEG550. The synthesis of DA12MPEG550 was performed following the same procedure as described for **7,** was used Poly (ethylene glycol) methyl ether (Mn 550) (0.2 mL, 0.35 mmol) and triethylamine (0.1 mL, 0.85 mmol) in 15 mL dry THF in 0 ˚C under a nitrogen atmosphere. To this solution, a solution of 3,5-bis-n-dodecylaminocarbonyl-benzoyl chloride (0.15 g, 0.28 mmol) in 5 mL of dry THF. The reaction was stirred overnight at room temperature under nitrogen atmosphere. Then, the solvent was removed under vacuum and the resulted crude product was purified with silica-gel ($CHCl_3$: MeOH; 100:7.5) obtain a yellowish oil (0.12 g, 41 %). IR (ATR) *ν*/cm^{-1}: 3295 (N-H, amide), 2918 (C – H alkyl), 2848 (C – H PEG), 1726 (C = O ester), 1648 (C = O amide), 1250 (C-CO), 1093 (C - O ester); ^{1}H NMR (400 MHz/$CDCl_3$) δ ppm: δ 8.61 (2H, s), 8.48 (1H, s), 6.82 (2H, N-H), 4.53(2H, t, J=4.8 Hz), 3.85 (2H, t, J=4.8Hz), 3.61 (40H, m), 3.46 (4H, q, J= 6.8 Hz), 3.36 (3H, s), 1.65 (4H, m), 1.26 (52 H, m), 0.88 (6H, t, J= 6.4 Hz); ^{13}C NMR(100 MHz, $CDCl_3$) δ ppm: 165.6, 165.3, 135.5, 130.9, 130.7, 129.7, 71.9, 70.7, 70.6, 70.5, 70.4, 69.0, 64.6, 59.0, 40.4, 31.9, 29.6, 29.5, 29.4, 29.3, 27.0, 22.6, 14.1; ESI-MS: *m/z* calculated for $[C_{58}H_{106}N_2O_{16}]^+$ = 1086.8 Da Found = 1086.57 Da.

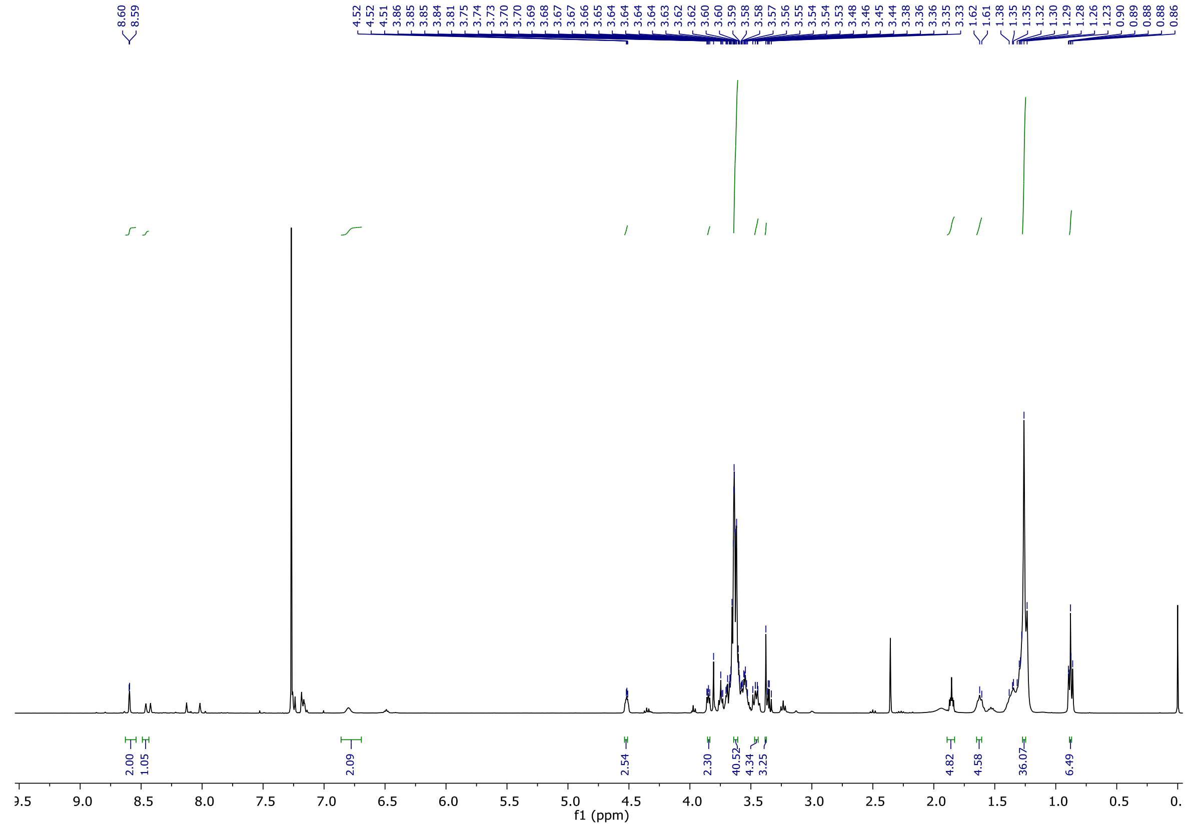

Figure S3: ^{1}H NMR spectrum of DA12MPEG550

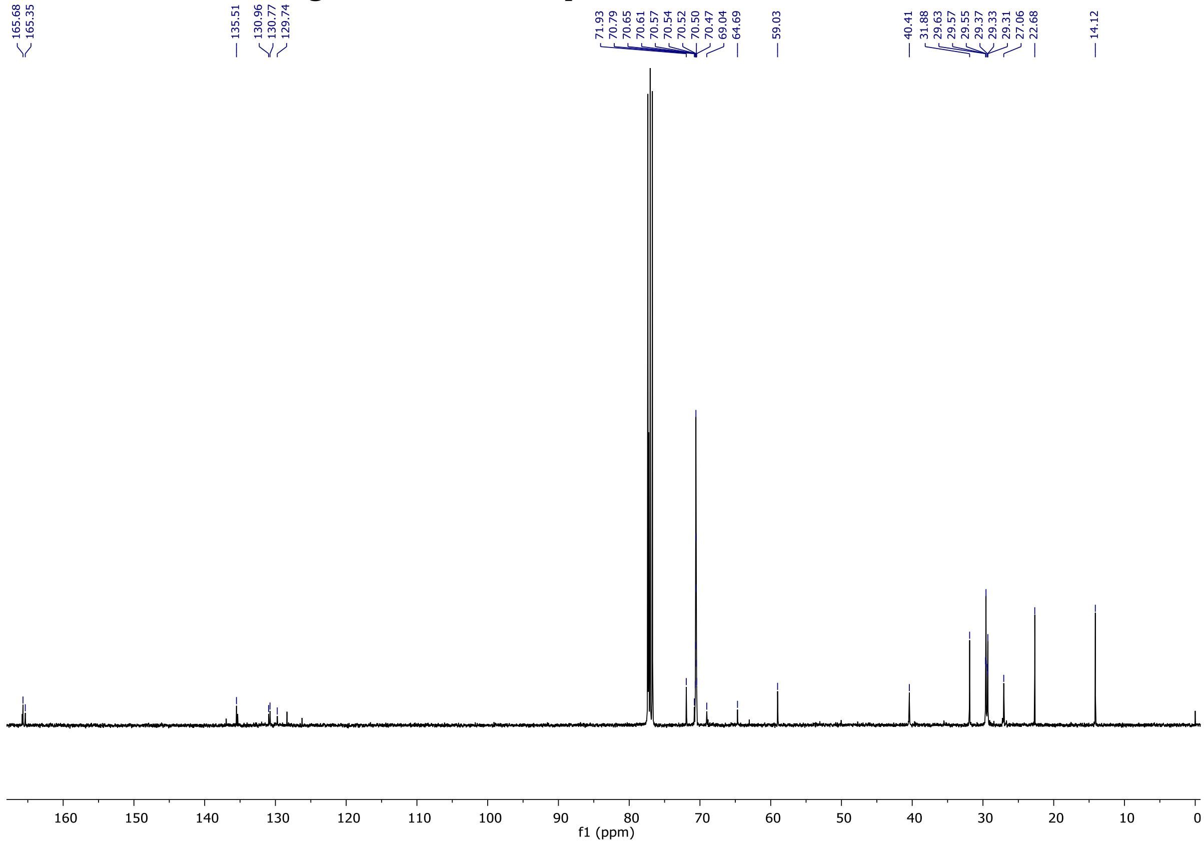

Figure S4: ^{13}C NMR spectrum of DA12MPEG550

DA16MPEG550. The synthesis of DA16MPEG550 was performed following the same procedure as described for **7,** was used Poly (ethylene glycol) methyl ether (Mn 550) (0.5 mL, 0.95 mmol) and triethylamine (0.25 mL, 1.86 mmol) in 15 mL dry THF in 0 ˚C under a nitrogen atmosphere. To this solution, a solution of 3,5-bis-n-hexadecylaminocarbonyl-benzoyl chloride (0.5 g, 0.75 mmol) in 5 mL of dry THF. The reaction was stirred overnight at room temperature under nitrogen atmosphere. Then, the solvent was removed under vacuum and the resulted crude product was purified with silica-gel ($CHCl_3$: MeOH; 100:8) obtain a colourless product (0.34 g, 40 %). IR (ATR) ν/cm^{-1}: 3295 (N-H, amide), 2918 (C – H alkyl), 2848 (C – H PEG), 1726 (C = O ester), 1648 (C = O amide), 1250 (C-CO), 1093 (C - O ester); ^{1}H NMR (400 MHz/$CDCl_3$) δ ppm: δ 8.61 (2H, s), 8.48 (1H, s), 6.82 (2H, N-H), 4.53(2H, t, J=4.8 Hz), 3.85 (2H, t, J=4.8Hz), 3.61 (40H, m), 3.46 (4H, q, J= 6.8 Hz), 3.36 (3H, s), 1.65 (4H, m), 1.26 (52 H, m), 0.88 (6H, t, J= 6.4 Hz); ^{13}C NMR(100 MHz, $CDCl_3$) δ ppm: 165.6, 165.3, 135.4,130.9, 130.8, 129.7, 129.0, 71.9, 70.7, 70.6, 70.5, 70.4, 70.3, 69.0, 64.6, 59.0, 40.4, 31.9, 29.7, 29.6, 29.5, 29.4, 29.3, 27.2, 27.0, 22.69, 14.1; ESI-MS: *m/z* calculated for $[C_{66}H_{122}N_2O_{16}]^+$ = 1215.88 Da Found = 1215.86 Da.

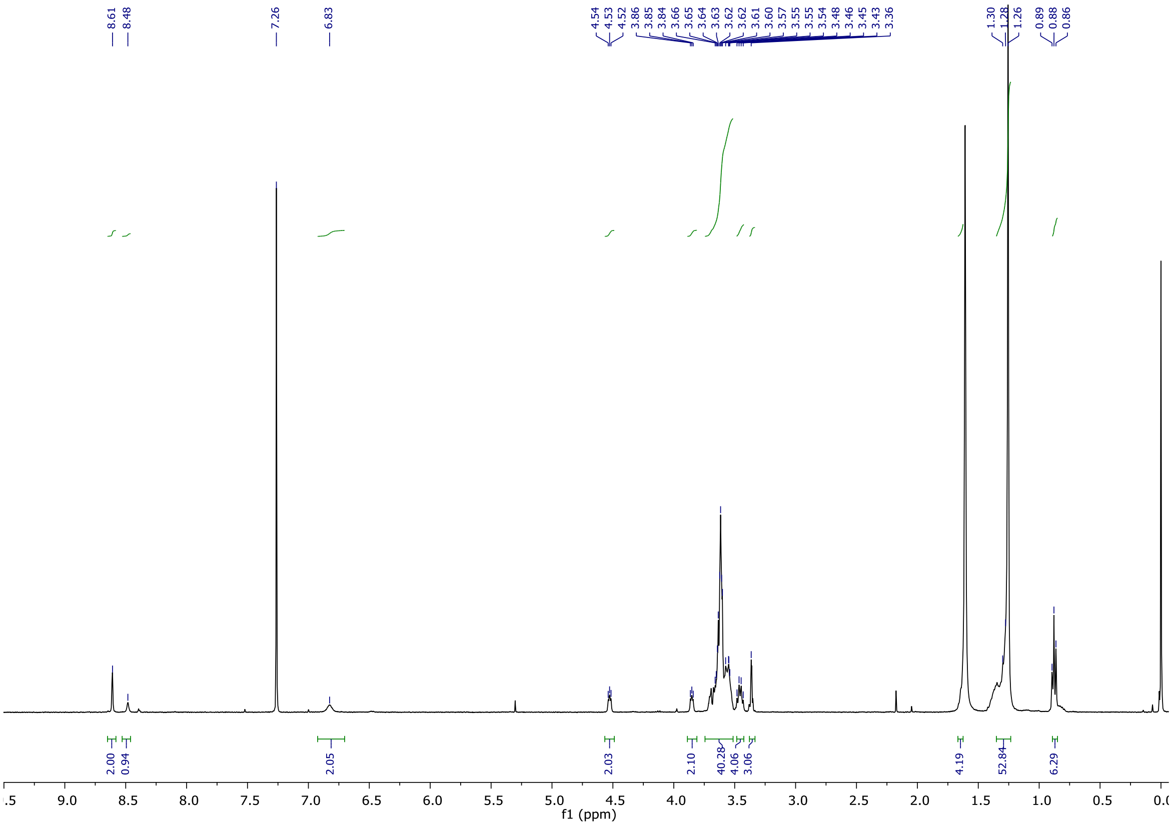

Figure S5: ^{1}H NMR spectrum of DA16MPEG550

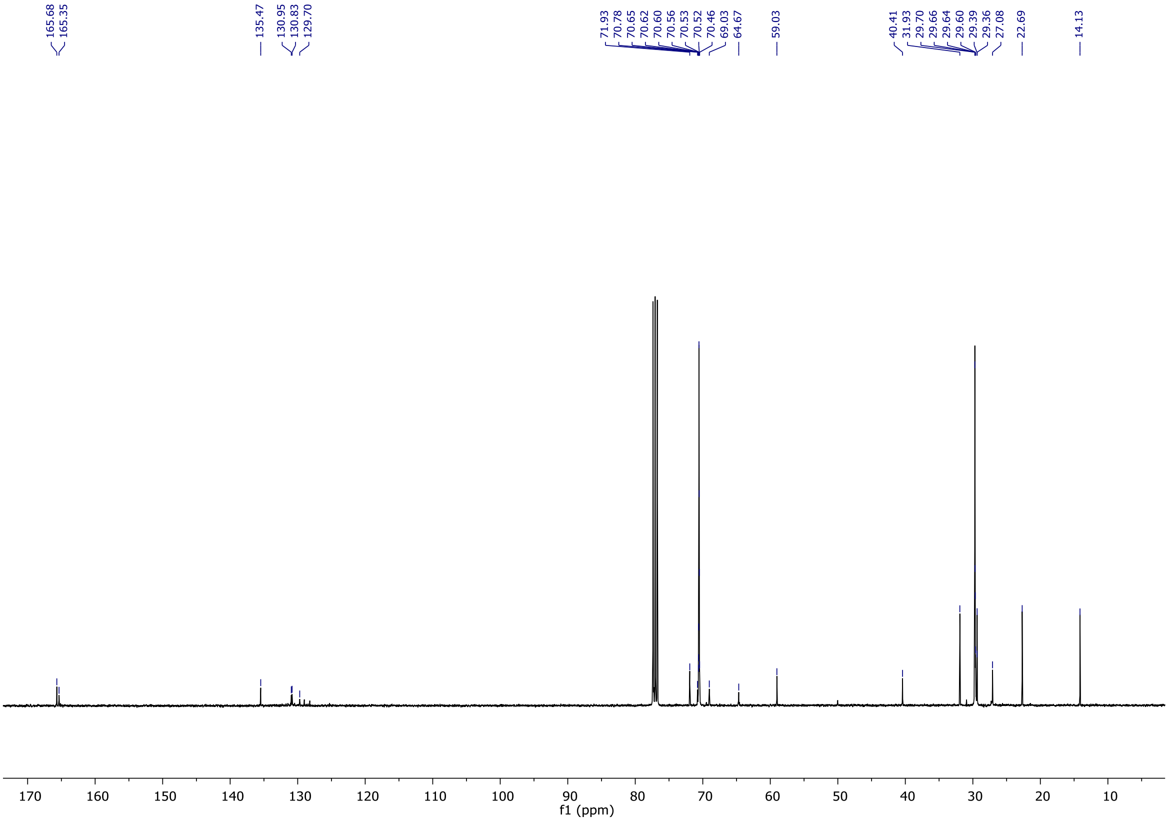

Figure S6: ^{13}C NMR spectrum of DA16MPEG550

DA18MPEG550. The synthesis of DA18MPEG550 was performed following the same procedure as described for **7,** was used Poly (ethylene glycol) methyl ether (Mn 550) (0.19 mL, 0.33 mmol) and triethylamine (0.1 mL, 0.84 mmol) in 15 mL dry THF in 0 ˚C under a nitrogen atmosphere. To this solution, a solution of 3,5-bis-n-octadecylaminocarbonyl-benzoyl chloride (0.2 g, 0.28 mmol) in 5 mL of dry THF. The reaction was stirred overnight at room temperature under nitrogen atmosphere. Then, the solvent was removed under vacuum and the resulted crude product was purified with silica-gel ($CHCl_3$: MeOH; 100:8) obtain a colourless product (0.13 g, 38 %). IR (ATR) *ν*/cm^{-1}: 3295 (N-H, amide), 2918 (C – H alkyl), 2848 (C – H PEG), 1726 (C = O ester), 1648 (C = O amide), 1250 (C-CO), 1093 (C - O ester); ^{1}H NMR (400 MHz/$CDCl_3$) δ ppm: δ 8.61 (2H, s), 8.48 (1H, s), 6.82 (2H, N-H), 4.53(2H, t, J=4.8 Hz), 3.85 (2H, t, J=4.8Hz), 3.61 (40H, m), 3.46 (4H, q, J= 6.8 Hz), 3.36 (3H, s), 1.65 (4H, m), 1.26 (52 H, m), 0.88 (6H, t, J= 6.4 Hz); ^{13}C NMR(100 MHz, $CDCl_3$) δ ppm: 165.6, 165.3, 135.4,130.9, 130.8, 129.7, 129.0, 71.9, 70.7, 70.6, 70.5, 70.4, 70.3, 69.0, 64.6, 59.0, 50.0, 40.4, 31.9, 29.7, 29.6, 29.5, 29.4, 29.3, 27.2, 27.0, 22.69, 14.1; ESI-MS: *m/z* calculated for $[C_{74}H_{138}N_2O_{16}]^+$ = 1311.00 Da Found = 1311.05 Da.

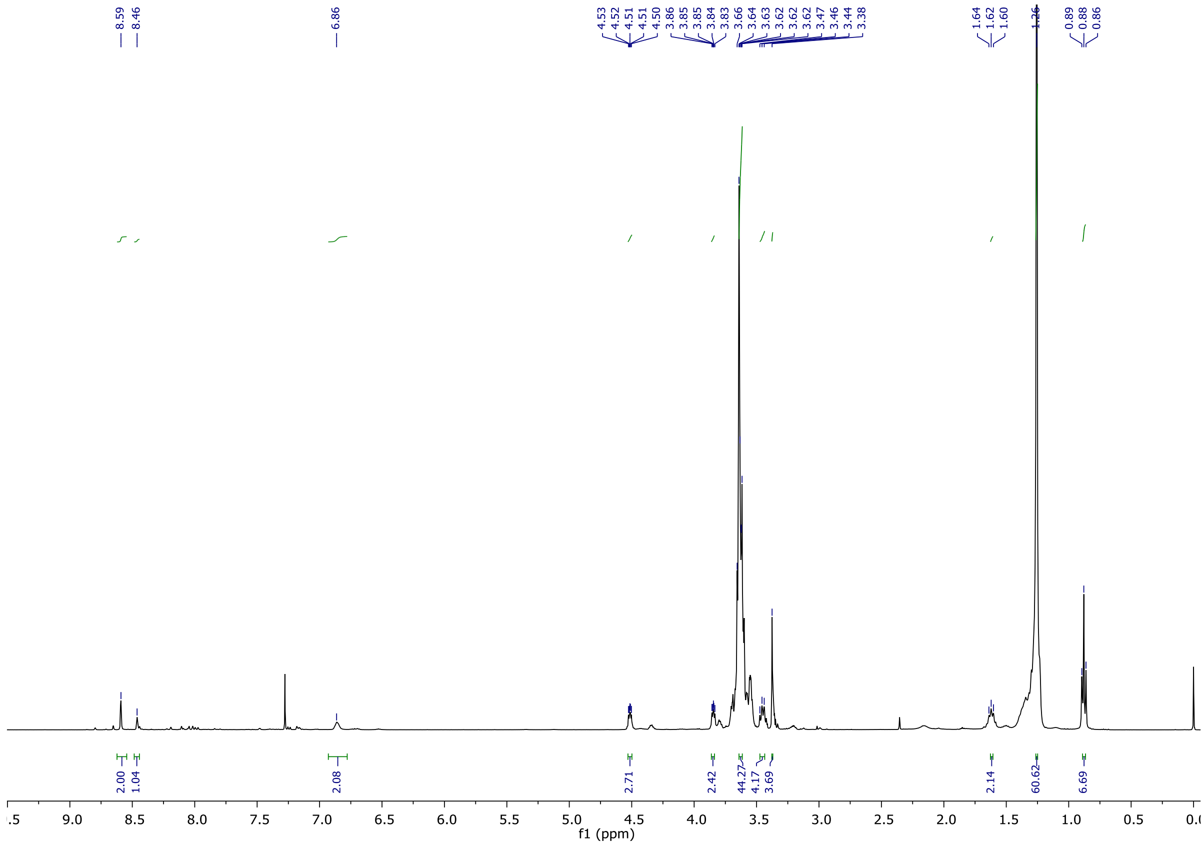

Figure S7: ^{1}H NMR spectrum of DA18MPEG550

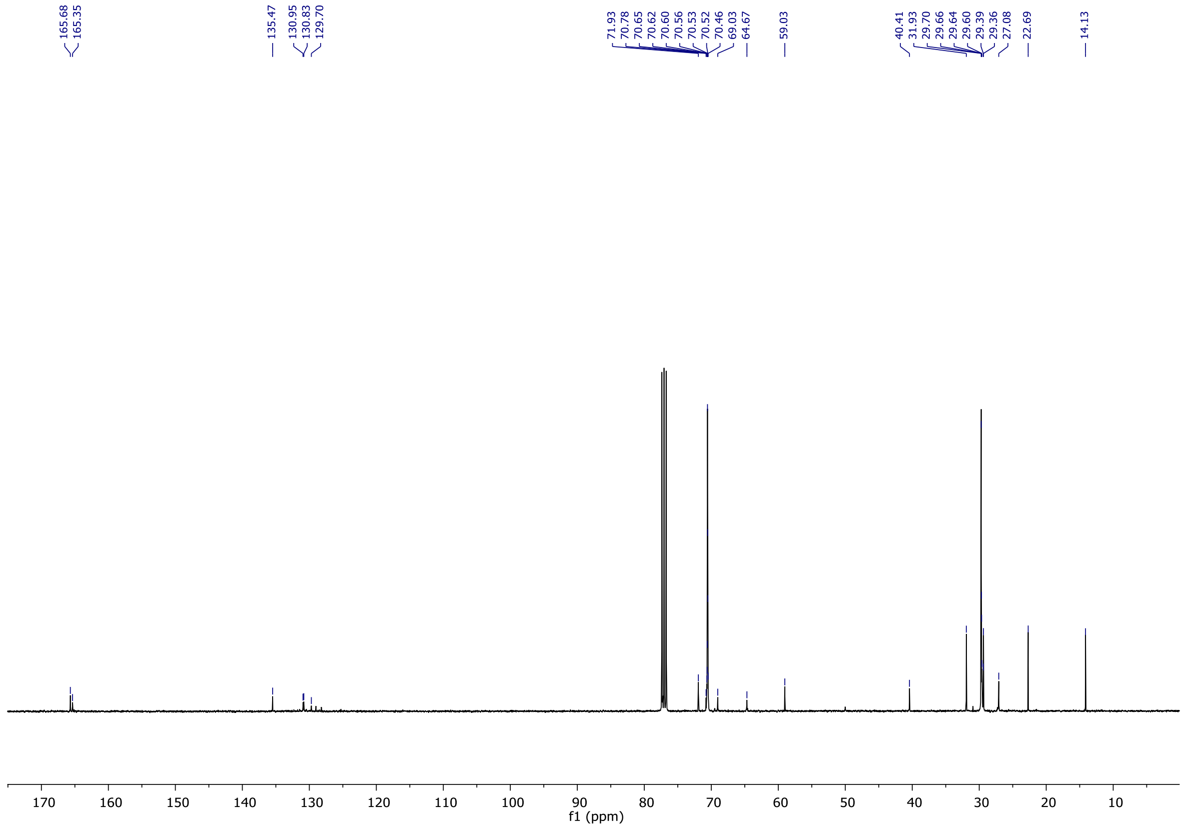

Figure S8: ^{13}C NMR spectrum of DA18MPEG550

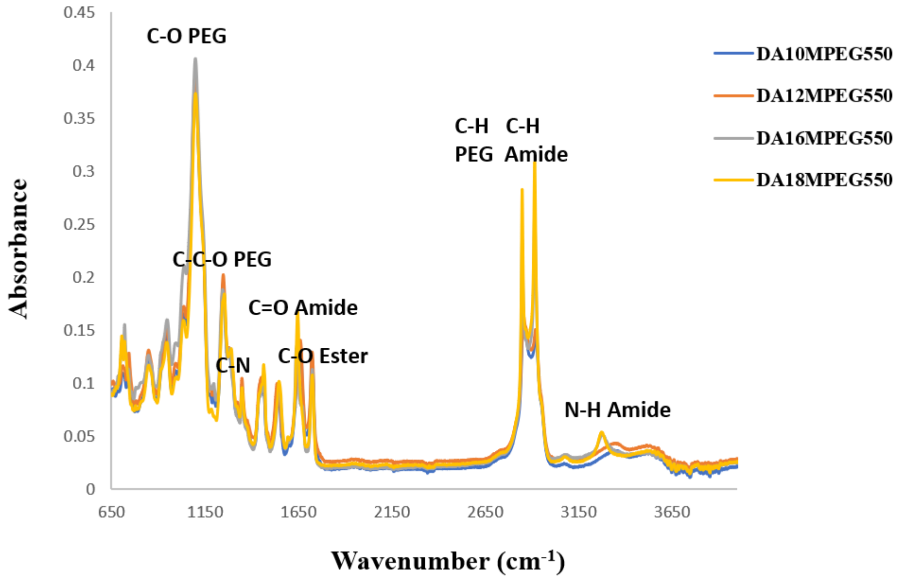

Figure S9: FT-IR spectra of DAmMPEG550.

Synthesis of MA10DPEG550, MA12DPEG550, MA16DPEG550 and MA18DPEG550

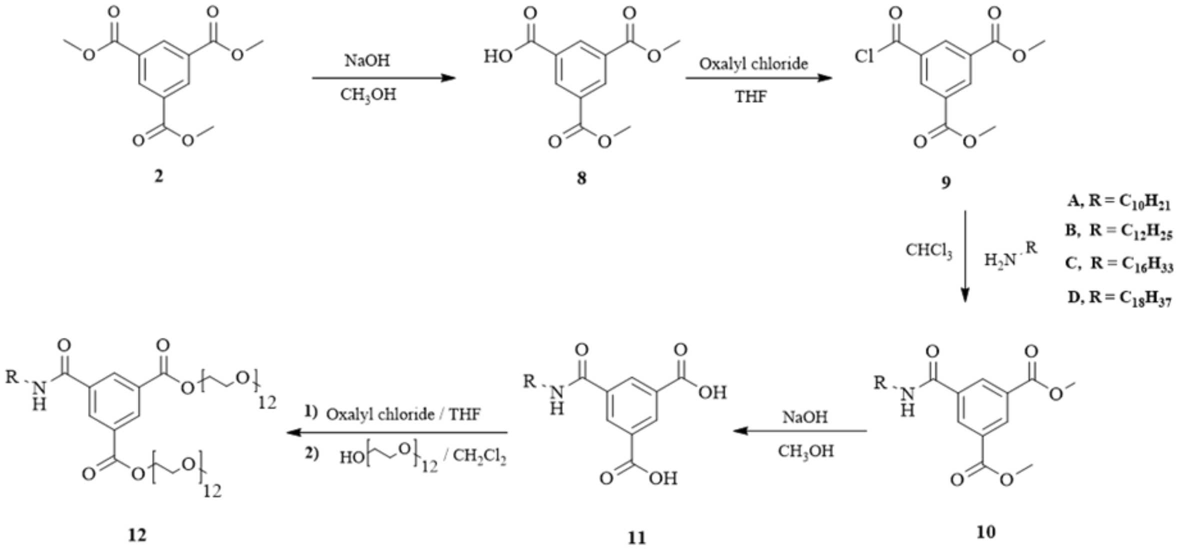

MAmDPEG550

Scheme S2. Reaction scheme for MAmDPEG550 compounds.

Synthesis of 1,3-dimethoxycarbonyl-benzene-5-carboxylic acid (8). Benzene-1,3,5-tricarboxylic acid trimethyl ester **2** (5.00 g, 19.82 mmol) and 4M NaOH (0.95 g, 23.78 mmol) were dissolved in methanol (150 mL) and refluxed for overnight at 85 ˚C. after which the solvent was removed under vacuum and dissolved in CH_2Cl_2 (300 mL) and extracted with saturated sodium bicarbonate solution (600 mL). The collected aqueous phase was washed with CH_2Cl_2 and acidified with 1M HCl resulting white precipitate that filtered and dried under vacuum giving white solid (3.30 g, 70%). IR (ATR) *ν*/cm^{-1}: 3248 (O-H, carboxylic acid), 1701 (C = O ester), 1215 (C - O ester), 1436 (C = C, Aromatic);^{1}H NMR (400 MHz, $CDCl_3$) δ ppm: 8.68 (3H, d, Ar–H), 3.96 (6H, s, –O*CH₃*); ^{13}C NMR (100 MHz, $CDCl_3$) δ ppm: 165.62, 165.40, 133.09, 132.03, 52.65; M.P. = 192-195 ˚C**;** ESI-MS: *m/z* calculated for $[C_{11}H_{10}O_6+H]^+$ = 239.19 Da Found = 239.05 Da.

Synthesis of dimethyl 5-(chlorocarbonyl) isophthalate (9). 1,3-dimethoxycarbonyl-benzene-5-carboxylic acid **8** (0.5 g, 2.10 mmol) was dissolved in 25 mL dry chloroform. After addition of a catalytic amount DMF, oxalyl chloride (190 µL, 2.25 mmol) in 10 mL dry chloroform was added drop wise. After stirring for 90 minutes, the excess oxalyl chloride and chloroform were removed *in vacuo*. The remaining solid was co-evaporated twice with 50 mL toluene to ensure the removal of oxalyl chloride. The product was obtained as a yellow oil (0.53 g, 98%); IR (ATR) *ν*/cm^{-1}: 1722 (C = O acid chloride), 1436 (C = C, Aromatic), 1267 (C - O), 731 (C - Cl); ^{13}C NMR (100 MHz, $CDCl_3$) δ ppm: 167.62, 165.65,133.84, 133.32, 132.21, 129.47, 52.70.

Synthesis of dimethyl 5-(alkylcarbamoyl)isophthalate (10). A solution of 1 eq. alkylamine and 1.3 eq. TEA was dissolved in dry CH_2Cl_2 under nitrogen atmosphere at 0˚C. To this solution, a solution of dimethyl 5-(chlorocarbonyl) isophthalate **9** (1 eq.) in dry CH_2Cl_2 was added drop wise to that and stirred for 24h at room temperature. Then, the solvent was removed under vacuum and the crude product was purified by column chromatography (CH_2Cl_2: MeOH, 100:1) yielding a white powder.

10A: (0.42 g, 71%); ; IR (ATR) *ν*/cm^{-1}: 2915 (N-H), 1731.2 (C=O, ester), 1629.4 (C=O, amide), 1559.8 (N-H), 1440.9 (C=C, Aromatic), 1267 (C-O, ester), 1178.7 (C-N, amide); ^{1}H-NMR (400 MHz, $CDCl_3$) δ ppm: 8.78 (1H, t, = 1.62 MHz), 8.60 (2H, d, J= 1.56 MHz), 6.30 (1H, t, N-*H*), 3.97 (6H, s, O-C*H₃*), 3.48 (2H, m, N-C*H₂*), 1.64 (2H, m, N-CH_2CH2), 1.26 (14H, m, alkyl), 0.87 (3H, t, J= 6.8 MHz, -C*H₃*); ^{13}C NMR(100 MHz, $CDCl_3$) δ ppm: 165.5, 165.4,135.6, 133.1, 132, 131.1, 52.6, 40.4, 31.9, 29.7, 29.6, 29.5, 29.4, 27.1, 22.6, 14.1; ESI-MS: *m/z* calculated for $[C_{21}H_{31}NO_5+H]^+$ = 378.48 Da Found = 378.22 Da.

10B: (0.43 g, 76%); ; IR (ATR) *ν*/cm^{-1}: 2913 (N-H), 1736.1 (C=O, ester), 1625.9 (C=O, amide), 1556.2 (N-H), 1438.9 (C=C, Aromatic), 1264 (C-O, ester), 1172.9 (C-N, amide); ^{1}H-NMR (400 MHz, $CDCl_3$) δ ppm: 8.78 (1H, t, = 1.62 MHz), 8.60 (2H, d, J= 1.56 MHz), 6.30 (1H, t, N-*H*), 3.97 (6H, s, O-C*H₃*), 3.48 (2H, m, N-C*H₂*), 1.64 (2H, m, N-CH_2CH2), 1.26 (18H, m, alkyl), 0.87 (3H, t, J= 6.8 MHz, -C*H₃*); ^{13}C NMR(100 MHz, $CDCl_3$) δ ppm: 165.5,

165.4,135.6, 133.1, 132, 131.1, 52.6, 40.4, 31.9, 29.7, 29.6, 29.5, 29.4, 27.1, 22.6, 14.1; ESI-MS: *m/z* calculated for $[C_{23}H_{35}NO_5+H]^+$ = 406.48 Da Found = 406.25 Da.

10C: (1.12 g, 63%)**;** IR (ATR) *ν*/cm^{-1}: 2917 (N-H), 2849.4 (C-H), 1733.8 (C=O, ester), 1637.6 (C=O, amide), 1534.8 (N-H), 1443.23 (C=C, Aromatic), 1247 (C-O, ester), 1195.8 (C-N, amide), 742.8 (N-H); ^{1}H-NMR (400 MHz, $CDCl_3$) δ ppm: 8.79 (1H, s, Aromatic), 8.61 (2H, s, Aromatic), 6.22 (1H, t, N-*H*), 3.97 (6H, s, O-C*H*$_3$), 3.49 (2H, m, N-C*H*$_2$), 1.64 (2H, m, N-CH_2CH2), 1.35 (26H, m, alkyl), 0.87 (3H, t,-C*H*$_3$); ^{13}C NMR(100 MHz, $CDCl_3$) δ ppm: 165.62, 165.27, 133.09, 132.03, 131.18, 52.65, 40.43, 31.94, 29.58, 22.70, 14.14; ESI-MS: *m/z* calculated for $[C_{27}H_{43}NO_5+H]^+$ = 462.64 Da Found = 462.64 Da.

10D: (0.50 g, 67%); ; IR (ATR) *ν*/cm^{-1}: 2921 (N-H), 1729.8 (C=O, ester), 1628.2 (C=O, amide), 1549.9 (N-H), 1436.7 (C=C, Aromatic), 1261 (C-O, ester), 1175 (C-N, amide); ^{1}H-NMR (400 MHz, $CDCl_3$) δ ppm: 8.78 (1H, t, = 1.62 MHz), 8.60 (2H, d, J= 1.56 MHz), 6.30 (1H, t, N-*H*), 3.97 (6H, s, O-C*H*$_3$), 3.48 (2H, m, N-C*H*$_2$), 1.64 (2H, m, N-CH_2CH2), 1.26 (30 H, m, alkyl), 0.87 (3H, t, J= 6.8 MHz, -C*H*$_3$); ^{13}C NMR(100 MHz, $CDCl_3$) δ ppm: 165.5, 165.4,135.6, 133.1, 132, 131.1, 52.6, 40.4, 31.9, 29.7, 29.6, 29.5, 29.4, 27.1, 22.6, 14.1; ESI-MS: *m/z* calculated for $[C_{29}H_{47}NO_5+H]^+$ = 490.70 Da Found = 490.35 Da.

Synthesis of 5-(alkylcarbamoyl) isophthalic (11). Dimethyl 5-(alkylcarbamoyl)isophthalate **10 (**1 eq.) was dissolved in methanol with 4M NaOH that was stirred and reflux at 85 °C for 24 hours. the mixture was cooled to room temperature before being poured to 1M HCl. A white powder precipitate was formed and filtered. The precipitate was recrystallized from ethyl acetate (2 times), leading to a white product.

11A: (0.12 g, 43%). IR (ATR) *ν*/cm^{-1}: 3329 (N – H amide), 2921 (O – H carboxylic acid), 1712 (C = O ester), 1628 (C = O amide), 1469 (C-N amide), 1152 (C - O ester); ^{1}H-NMR (400 MHz, DMSO-d_6) δ ppm: 8.87 (1H, t, = 1.62 MHz), 8.62 (2H, d, J= 1.68 MHz), 8.56 (1H, t, J= 1.66 MHz), 3.27 (2H, m), 1.50 (2H, m), 1.23 (14 H, m), 0.83 (3H, t, J= 6.8 MHz); ^{13}C NMR(100 MHz, DMSO-d_6) δ ppm: 165.5, 165.4,135.6, 133.1, 132, 131.1, 40.4, 31.9, 29.7, 29.6, 29.5, 29.4, 22.6, 14.1**;** ESI-MS: *m/z* calculated for $[C_{19}H_{27}NO_5+H]^+$ = 350.43 Da Found = 350.19 Da.

11B: (0.17 g, 48%). IR (ATR) *ν*/cm^{-1}: 3323 (N – H amide), 2918 (O – H carboxylic acid), 1721 (C = O ester), 1632 (C = O amide), 1461 (C-N amide), 1160 (C - O ester); ^{1}H-NMR (400 MHz, DMSO-d_6) δ ppm: 8.87 (1H, t, = 1.62 MHz), 8.62 (2H, d, J= 1.68 MHz), 8.56 (1H, t, J= 1.66 MHz), 3.27 (2H, m), 1.50 (2H, m), 1.23 (18 H, m), 0.83 (3H, t, J= 6.8 MHz); ^{13}C NMR(100 MHz, DMSO-d_6) δ ppm: 165.5, 165.4,135.6, 133.1, 132, 131.1, 40.4, 31.9, 29.7, 29.6, 29.5, 29.4, 22.6, 14.1**;** ESI-MS: *m/z* calculated for $[C_{21}H_{31}NO_5+H]^+$ = 378.48 Da Found = 378.22 Da.

11C: (0.30 g, 42%). IR (ATR) *ν*/cm^{-1}: 3358 (N – H amide), 2917 (O – H carboxylic acid), 1704 (C = O ester), 1630 (C = O amide), 1472 (C-N amide), 1158 (C - O ester); ^{1}H-NMR (400 MHz, DMSO-d_6) ppm δ: 13.52 (1H, OH), 8.89 (1H, t, N*H*), 8.63 (3H, d, Aromatic), 1.57 (2H, m, N-*CH*$_2$), 1.32 (26H, s, CH_2*CH*$_2$), 0.87 (3H, t, -*CH*$_3$); ^{13}C NMR (400 MHz, DMSO-d_6) ppm

δ: 165.59, 164.72, 133.67, 133.09, 130.86, 40.43, 31.94, 29.19, 22.70, 14.14**;** ESI-MS: *m/z* calculated for $[C_{27}H_{43}NO_5+H]^+$ = 434.59 Da Found = 434.28 Da.

11D: (0.25 g, 57%). IR (ATR) *ν*/cm^{-1}: 3326 (N – H amide), 2923 (O – H carboxylic acid), 1719 (C = O ester), 1629 (C = O amide), 1457 (C-N amide), 1164 (C - O ester); ^{1}H-NMR (400 MHz, DMSO-d_6) δ ppm: 8.87 (1H, t, = 1.62 MHz), 8.62 (2H, d, J= 1.68 MHz), 8.56 (1H, t, J= 1.66 MHz), 3.27 (2H, m), 1.50 (2H, m), 1.23 (30 H, m), 0.83 (3H, t, J= 6.8 MHz); ^{13}C NMR(100 MHz, DMSO-d_6) δ ppm: 165.5, 165.4,135.6, 133.1, 132, 131.1, 40.4, 31.9, 29.7, 29.6, 29.5, 29.4, 22.6, 14.1**;** ESI-MS: *m/z* calculated for $[C_{27}H_{43}NO_5+H]^+$ = 462.64 Da Found = 462.31 Da.

General Synthesis for of MAmDPEG550 (12). As a first step 5-(alkylcarbamoyl)isophthaloyl dichloride was synthesised following the same procedure as described for **9**, 1 eq. of 5-(alkylcarbamoyl) isophthalic **11** and 2.5 eq. of oxalylchloride yielding a yellowish product. The resulted product was dissolved in dry THF and added slowly to a solution of 2 eq. Poly (ethylene glycol) methyl ether (Mn 550) and triethylamine 3 eq. in dry THF in ice bath under a nitrogen atmosphere. The reaction was stirred overnight at room temperature under nitrogen atmosphere. Then, the solvent was removed under vacuum and the resulting crude product was purified by column chromatography ($CHCl_3$: MeOH).

MA10DPEG550: MA10DPEG550 was performed following the same procedure as described for **12,** Poly (ethylene glycol) methyl ether (Mn 550) (0.50 mL, 0.90 mmol) and triethylamine (1.2 mL, 1.23 mmol) in 15 mL dry THF in 0 ˚C under a nitrogen atmosphere. To this solution, a solution of 5-(decylcarbamoyl)isophthaloyl dichloride (0.15 g, 0.40 mmol) in 5 mL of dry THF. The reaction was stirred overnight at room temperature under nitrogen atmosphere. Then, the solvent was removed under vacuum and the resulting crude product was purified with silica-gel ($CHCl_3$: MeOH; 100:4.5) providing a colourless oil (0.23 g, 42 %). IR (ATR) *ν*/cm^{-1}: 3295 (N-H, amide), 2925 (C – H alkyl), 2864 (C – H PEG), 1725 (C = O ester), 1636 (C = O amide), 1248(C-CO), 1099 (C - O ester) ; ^{1}H NMR (400 MHz/$CDCl_3$) δ ppm: δ 8.80 (1H, s), 8.64 (2H, s), 6.70 (1H, N-H), 4.52 (4H,t, J= 5 Hz), 3.85 (4H,t, J= 5 Hz), 3.64 (111H, m), 3.55(4H, t, J= 4.8), 3.46 (2H, q, J= 6.8 Hz), 3.38 (6H, s), 1.62 (2H, m), 1.26 (14 H, m), 0.88 (3H, t, J= 6.8 Hz^{13}C NMR(100 MHz, $CDCl_3$) δ ppm: 165.4, 165.1, 135.6,133.2, 132.4, 131.0, 71.9, 70.7, 70.6, 70.5, 70.4, 70.3, 69.0, 64.7, 59.0,40.4, 31.9, 29.8, 29.7, 29.6, 29.5, 29.4, 29.3, 28.2, 27.0, 22.7, 14.1; ESI-MS: *m/z* calculated for $[C_{69}H_{127}NO_{29}]^+$ = 1433.85 Da Found = 1433.94 Da.

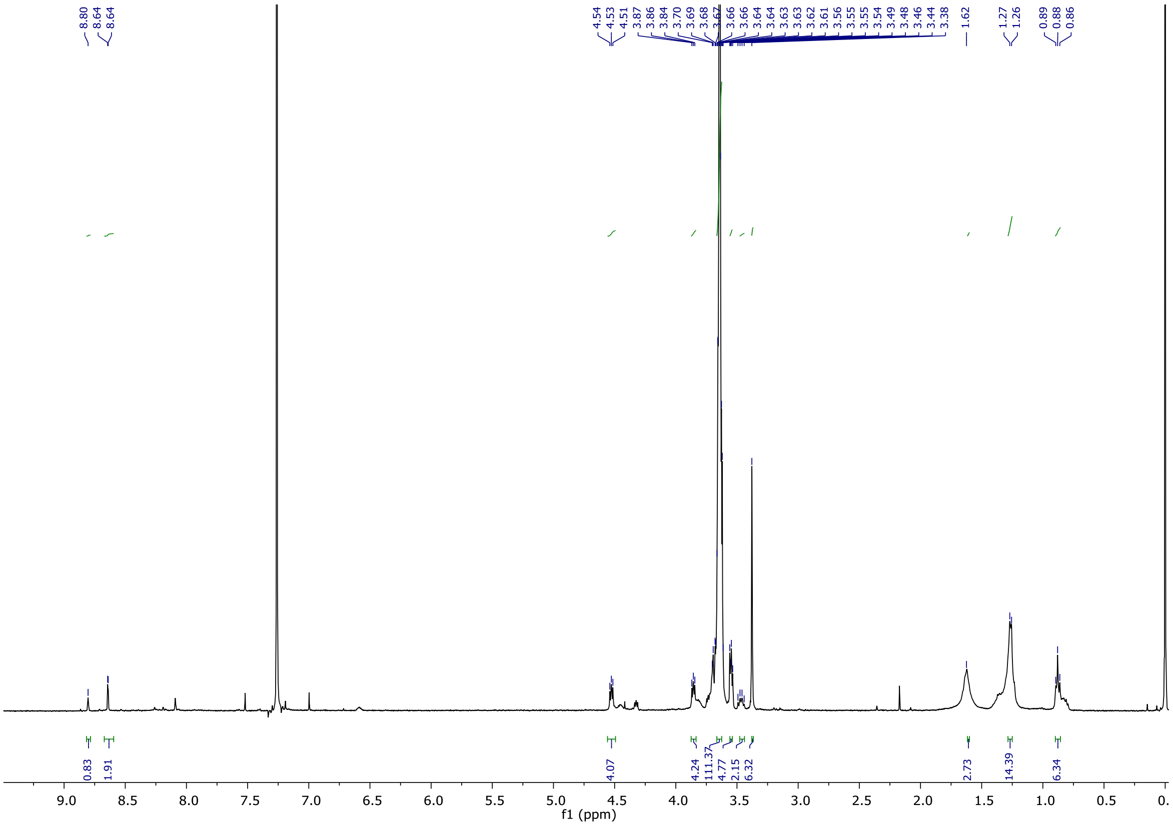

Figure S10: ^{1}H NMR spectrum of MA10DPEG550

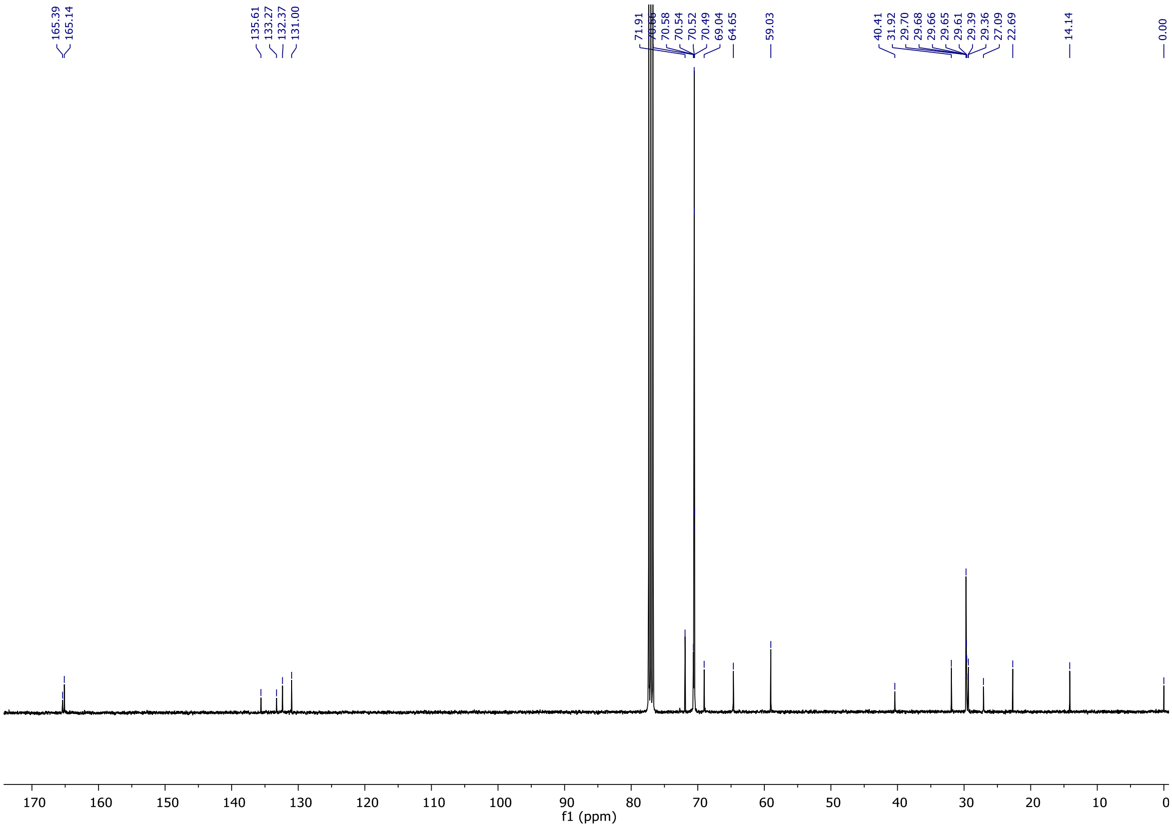

Figure S11: ^{13}C NMR spectrum of MA10DPEG550

MA12DPEG550: MA12DPEG550 was performed following the same procedure as described for **12,** Poly (ethylene glycol) methyl ether (Mn 550) (0.6 mL, 1.06 mmol) and triethylamine (0.2 mL, 1.93 mmol) in 15 mL dry THF in 0 ˚C under a nitrogen atmosphere. To this solution, a solution of 5-(dodecylcarbamoyl)isophthaloyl dichloride (0.2 g, 0.48 mmol) in 5 mL of dry THF. The reaction was stirred overnight at room temperature under nitrogen atmosphere. Then, the solvent was removed under vacuum and the resulting crude product was purified with silica-gel ($CHCl_3$: MeOH; 100:4.5) providing a colourless oil (0.27 g, 39 %). IR (ATR) ν/cm^{-1}: 3295 (N-H, amide), 2925 (C – H alkyl), 2864 (C – H PEG), 1725 (C = O ester), 1636 (C = O amide), 1248(C-CO), 1099 (C - O ester) ; ^{1}H NMR (400 MHz/$CDCl_3$) δ ppm: δ 8.80 (1H, s), 8.65 (2H, s), 6.70 (1H, N-H), 4.53 (4H,t, J= 4.8 Hz), 3.85 (4H,t, J= 4.8 Hz), 3.64 (111H, m), 3.55(4H, t, J= 4.8), 3.46 (2H, q, J= 6.7 Hz), 3.38 (6H, s), 1.62 (2H, m), 1.26 (26 H, m), 0.88 (3H, t, J= 6.6 Hz); ^{13}C NMR(100 MHz, $CDCl_3$) δ ppm: 165.4, 165.1, 135.6,133.2, 132.4, 131.0, 71.9, 70.7, 70.6, 70.5, 70.4, 70.3, 69.0, 64.7, 59.0,40.4, 31.9, 29.8, 29.7, 29.6, 29.5, 29.4, 29.3, 28.2, 27.0, 22.7, 14.1; ESI-MS: *m/z* calculated for $[C_{71}H_{131}NO_{29}]^+$ = 1461.88 Da Found = 1461.97 Da.

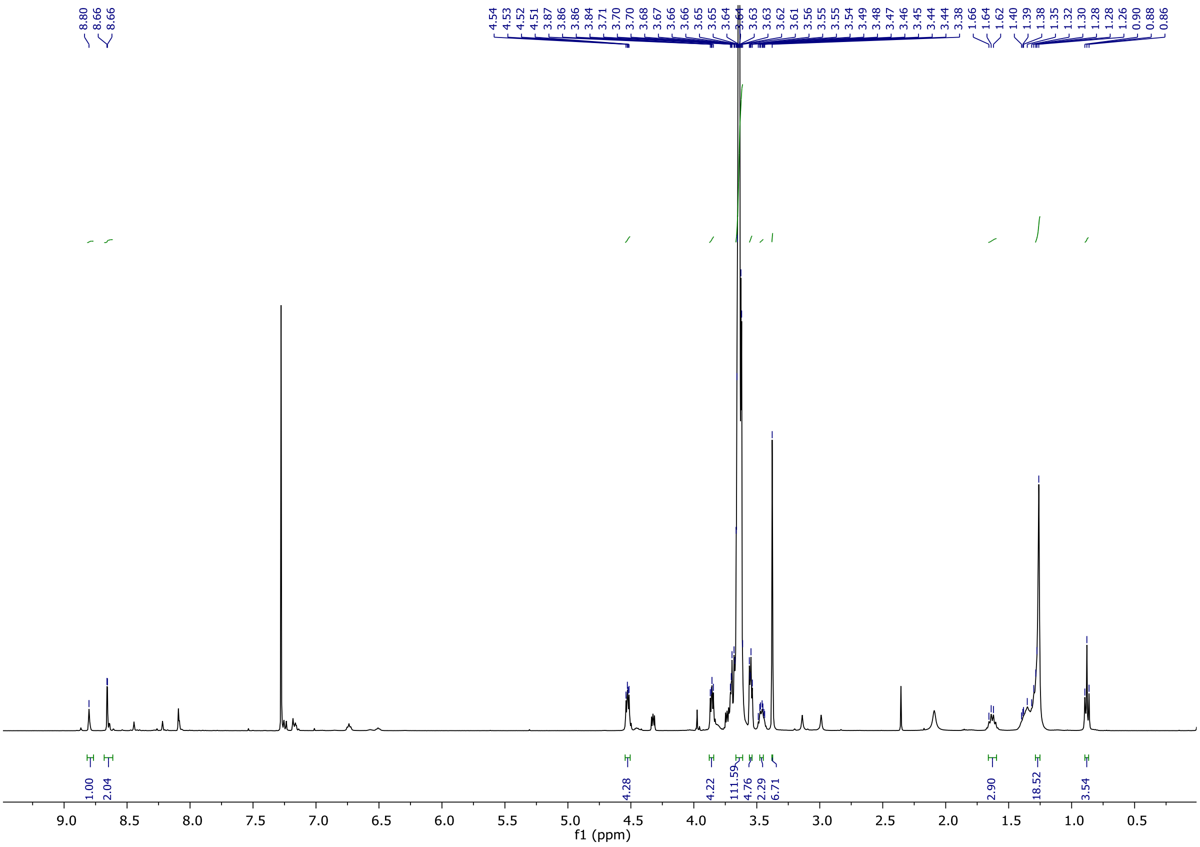

Figure S12: ^{1}H NMR spectrum of MA12DPEG550

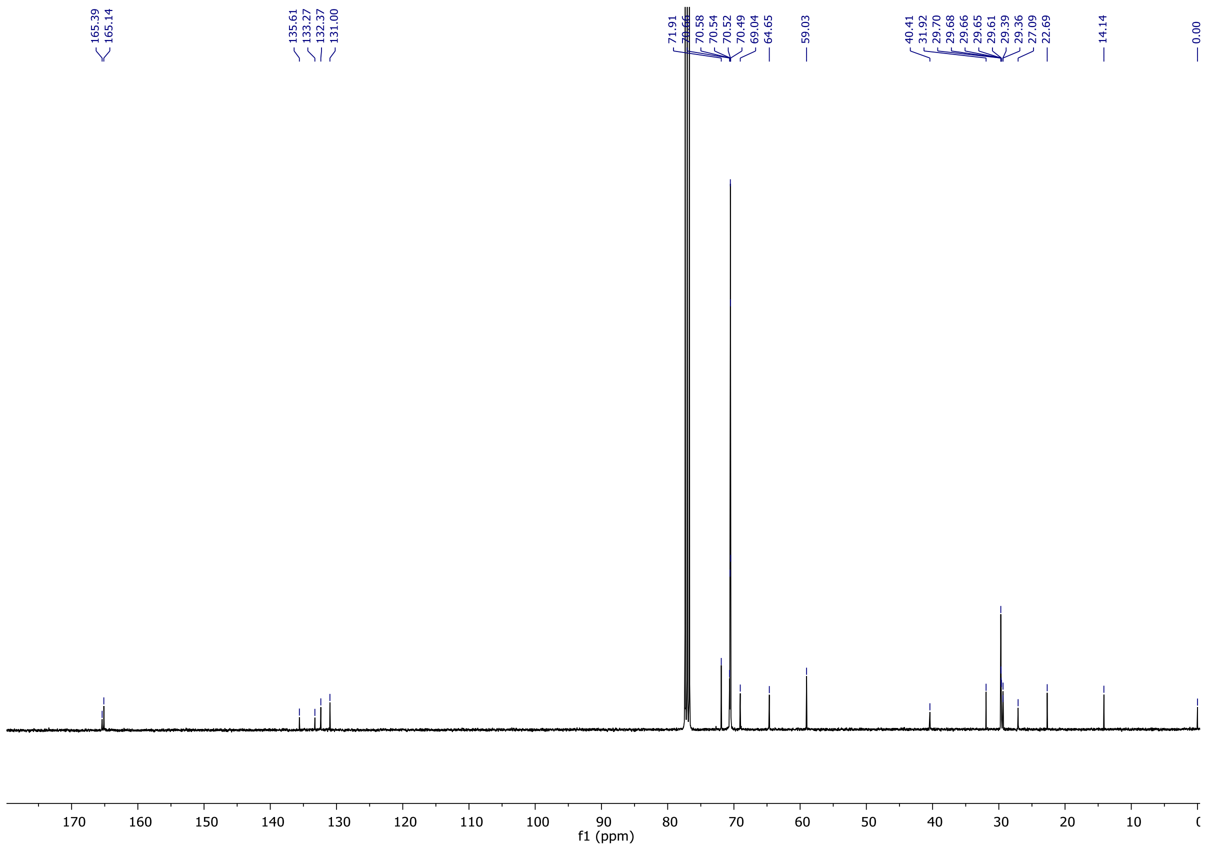

Figure S13: ^{13}C NMR spectrum of MA12DPEG550

MA16DPEG550: MA16DPEG550 was performed following the same procedure as described for **12,** Poly (ethylene glycol) methyl ether (Mn 550) (0.9 mL, 1.73 mmol) and triethylamine (0.33 mL, 2.60 mmol) in 15 mL dry THF in 0 ˚C under a nitrogen atmosphere. To this solution, a solution of 5-(hexadecylcarbamoyl)isophthaloyl dichloride (0.37g, 0.79 mmol) in 5 mL of dry THF. The reaction was stirred overnight at room temperature under nitrogen atmosphere. Then, the solvent was removed under vacuum and the resulting crude product was purified with silica-gel ($CHCl_3$: MeOH; 100:5) providing a yellowish oil (0.48 g, 41%). IR (ATR) ν/cm^{-1}: 3295 (N-H, amide), 2925 (C – H alkyl), 2864 (C – H PEG), 1725 (C = O ester), 1636 (C = O amide), 1248(C-CO), 1099 (C - O ester) ; ^{1}H NMR (400 MHz/$CDCl_3$) δ ppm: δ 8.81 (1H, s), 8.65 (2H, s), 6.70 (1H, N-H), 4.53 (4H,t, J= 4.84 Hz), 3.85 (4H,t, J= 4.88 Hz), 3.64 (111H, m), 3.55(4H, t, J= 4.74), 3.46 (2H, q, J= 6.78 Hz), 3.38 (6H, s), 1.62 (2H, m), 1.26 (26 H, m), 0.88 (3H, t, J= 6.62 Hz); ^{13}C NMR(100 MHz, $CDCl_3$) δ ppm: 165.4, 165.1, 135.6,133.2, 132.4, 131.0, 71.9, 70.7, 70.6, 70.5, 70.4, 70.3, 69.0, 64.7, 59.0,40.4, 31.9, 29.8, 29.7, 29.6, 29.5, 29.4, 29.3, 28.2, 27.0, 22.7, 14.1; ESI-MS: *m/z* calculated for $[C_{75}H_{139}NO_{29}]^+$ = 1552.08 Da Found = 1552.29 Da.

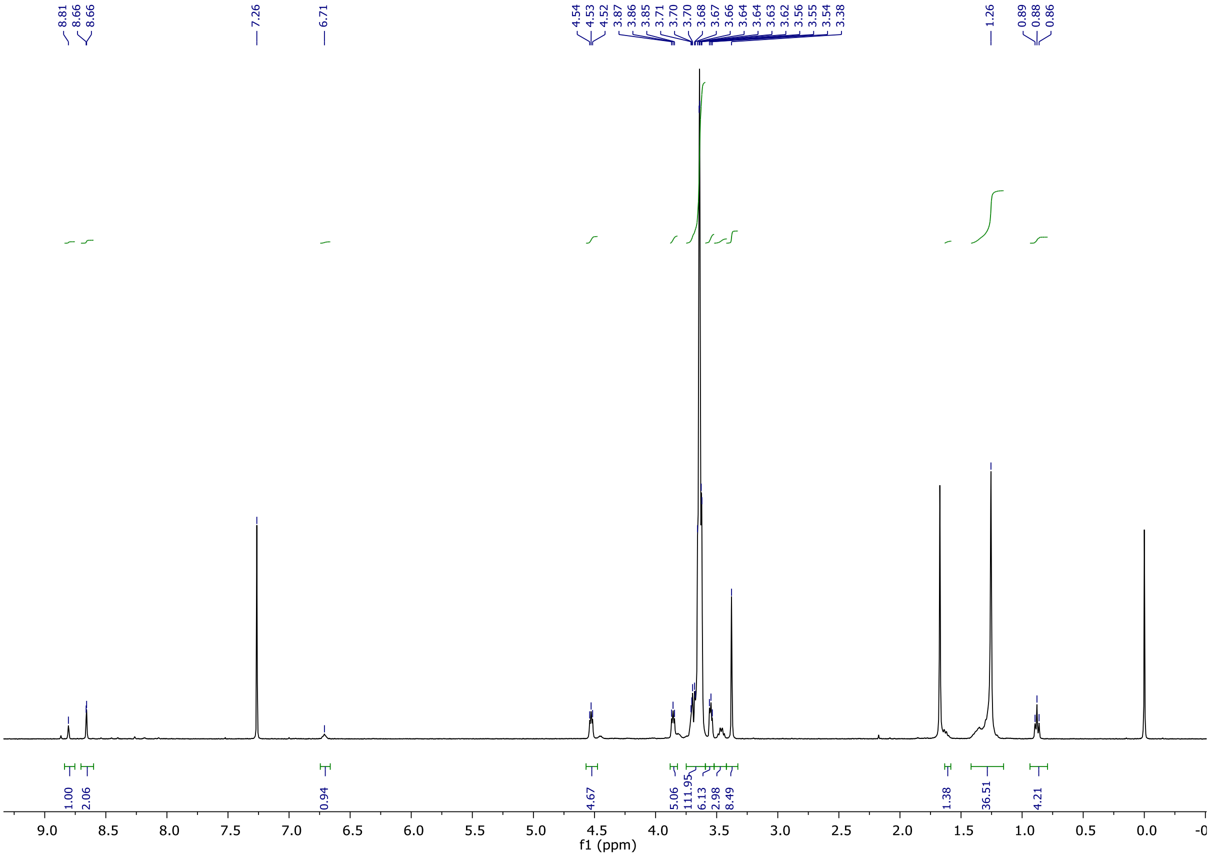

Figure S14: ^{1}H NMR spectrum of MA16DPEG550

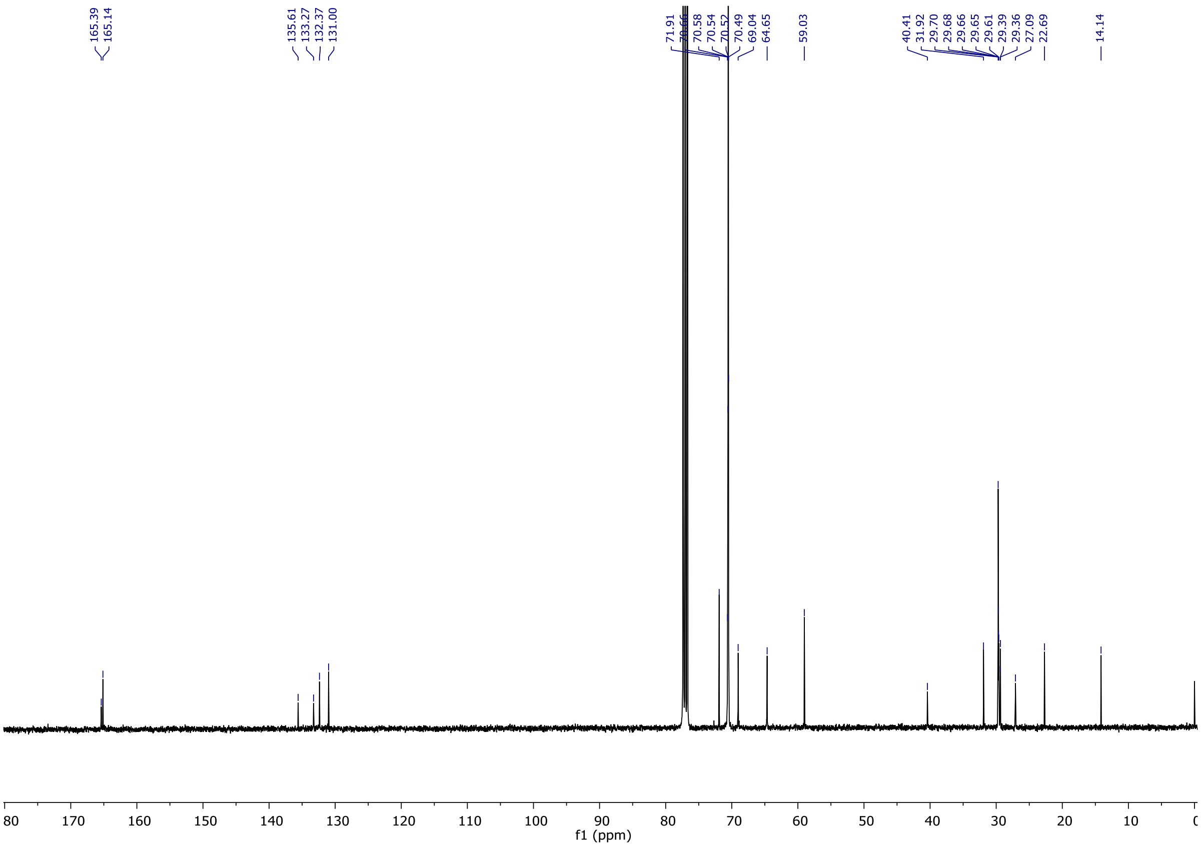

Figure S15: ^{13}C NMR spectrum of MA16DPEG550

MA18DPEG550: MA18DPEG550 was performed following the same procedure as described for **12,** Poly (ethylene glycol) methyl ether (Mn 550) (0.6 mL, 1.12 mmol) and triethylamine (0.28 mL, 2.0 mmol) in 15 mL dry THF in 0 ˚C under a nitrogen atmosphere. To this solution, a solution of 5-(octadecylcarbamoyl)isophthaloyl dichloride (0.25 g, 0.51 mmol) in 5 mL of dry THF. The reaction was stirred overnight at room temperature under nitrogen atmosphere. Then, the solvent was removed under vacuum and the resulting crude product was purified with silica-gel ($CHCl_3$: MeOH; 100:5.5) providing a yellowish oil (0.31 g, 40 %). IR (ATR) ν/cm^{-1}: 3295 (N-H, amide), 2925 (C – H alkyl), 2864 (C – H PEG), 1725 (C = O ester), 1636 (C = O amide), 1248(C-CO), 1099 (C - O ester) ; ^{1}H NMR (400 MHz/$CDCl_3$) δ ppm: δ 8.80 (1H, s), 8.65 (2H, d, J= 1.6), 6.70 (1H, N-H), 4.53 (4H,t, J= 4.84 Hz), 3.85 (4H,t, J= 4.88 Hz), 3.64 (111H, m), 3.55(4H, t, J= 4.74), 3.46 (2H, q, J= 6.78 Hz), 3.38 (6H, s), 1.62 (2H, m), 1.26 (30 H, m), 0.88 (3H, t, J= 6.62 Hz); ^{13}C NMR(100 MHz, $CDCl_3$) δ ppm: 165.4, 165.1, 135.6,133.2, 132.4, 131.0, 71.9, 70.6, 70.5, 70.4, 70.3, 69.0, 59.0, 40.4, 40.2, 31.9, 29.8, 29.7, 29.6, 29.5, 29.4, 29.3, 29.2, 27.0, 22.7, 14.1; ESI-MS: *m/z* calculated for $[C_{77}H_{143}NO_{29}]^+$ = 1545.97 Da Found = 1546.06 Da.

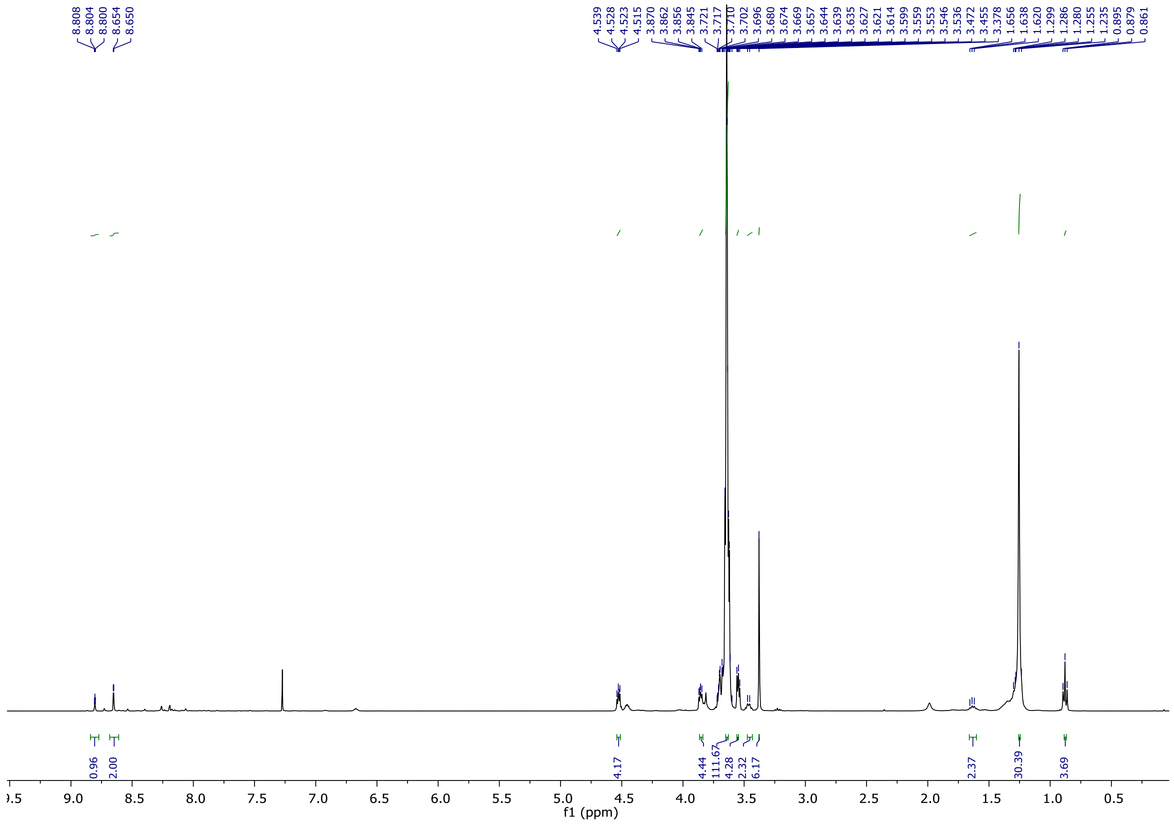

Figure S16: H NMR spectrum of MA18DPEG550

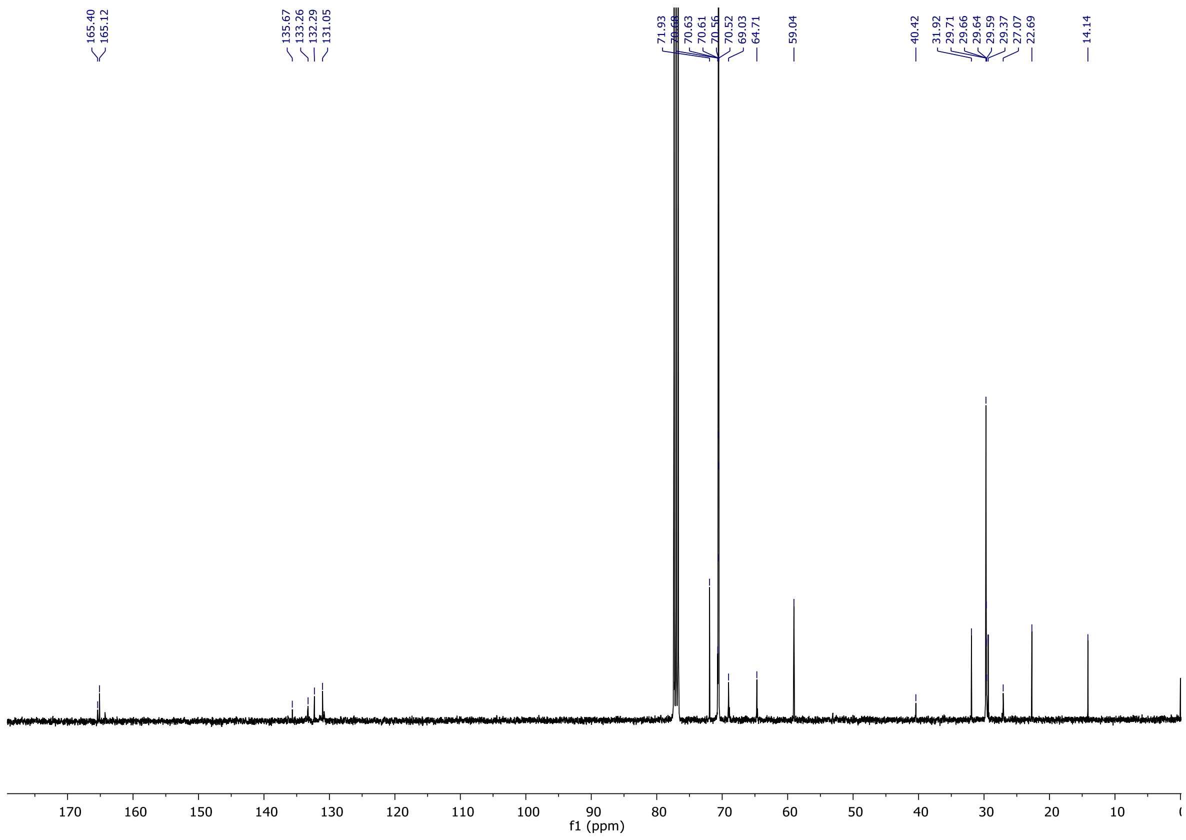

Figure S17: ^{13}C NMR spectrum of MA18DPEG550

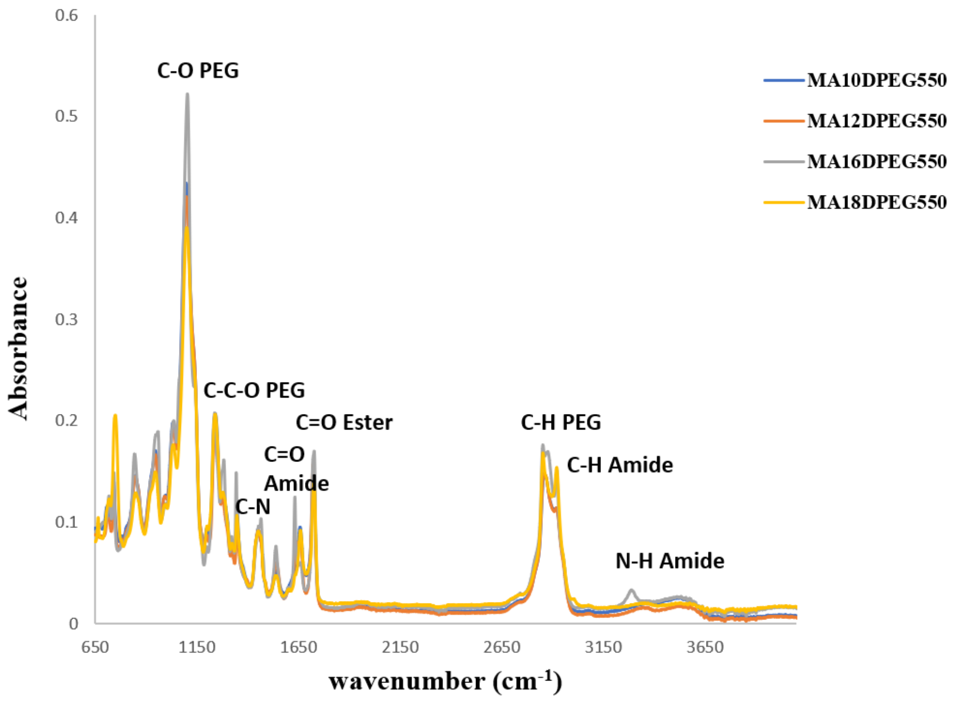

Figure S18: FT-IR spectrum of MAmDPEG550

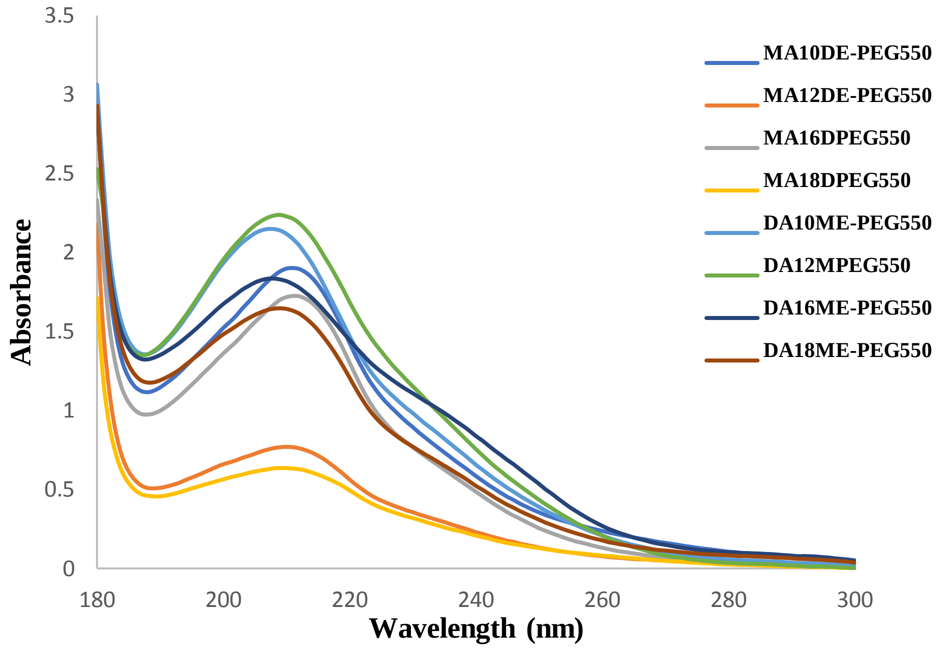

Figure S19: UV spectra for DAmMPEG550 and MAmDPEG550 series

(a)

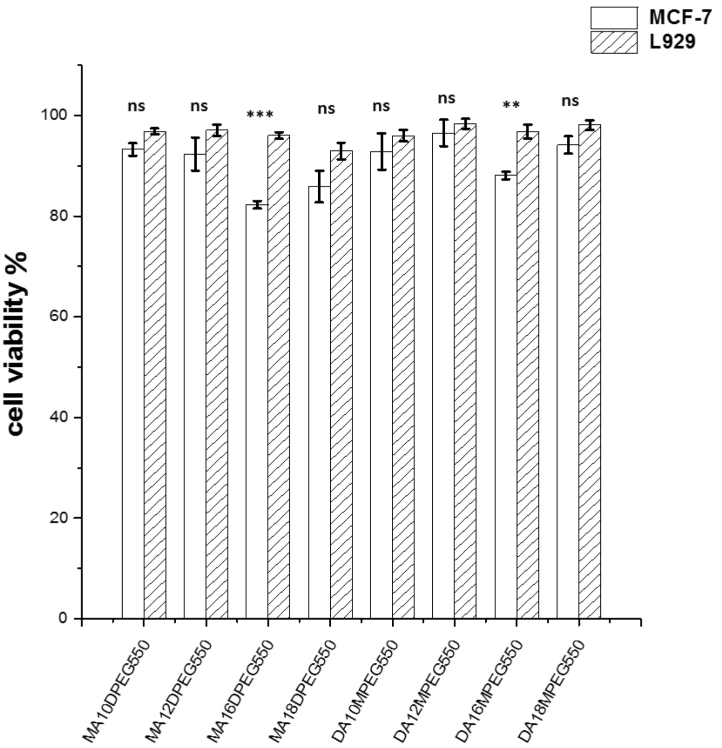

(b)

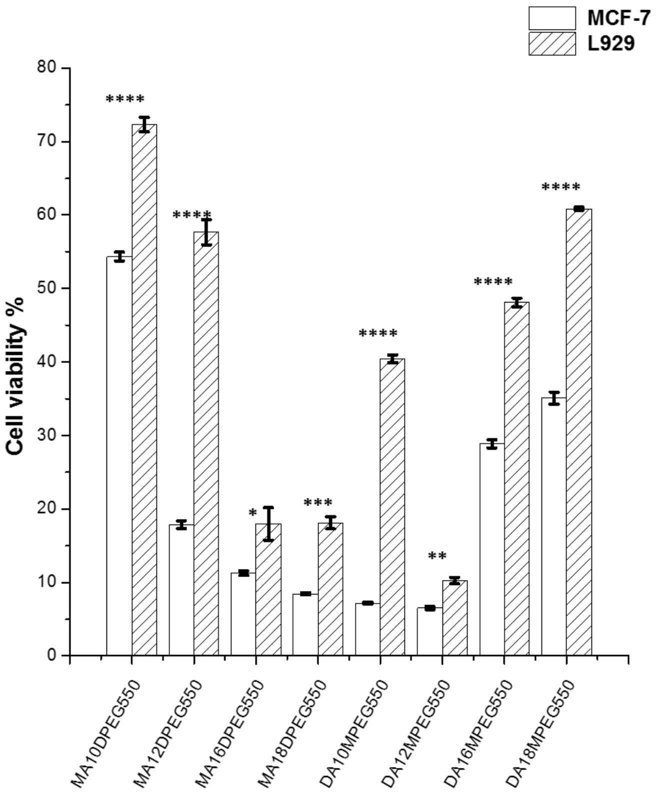

Figure S20: Cell viabilities from MTT (3-(4,5-dimethylthiazol-2-yl)-2,5-diphenyltetrazolium bromide) assays for L929 fibroblast and MCF-7 breast cancer cell lines for the compounds indicated (a) 1 μM, (b) 100 μM. Student's *t*-test significance values $p < 0.05$ *, $p < 0.01$ **, $p < 0.001$ ***, $p < 0.0001$ ****.

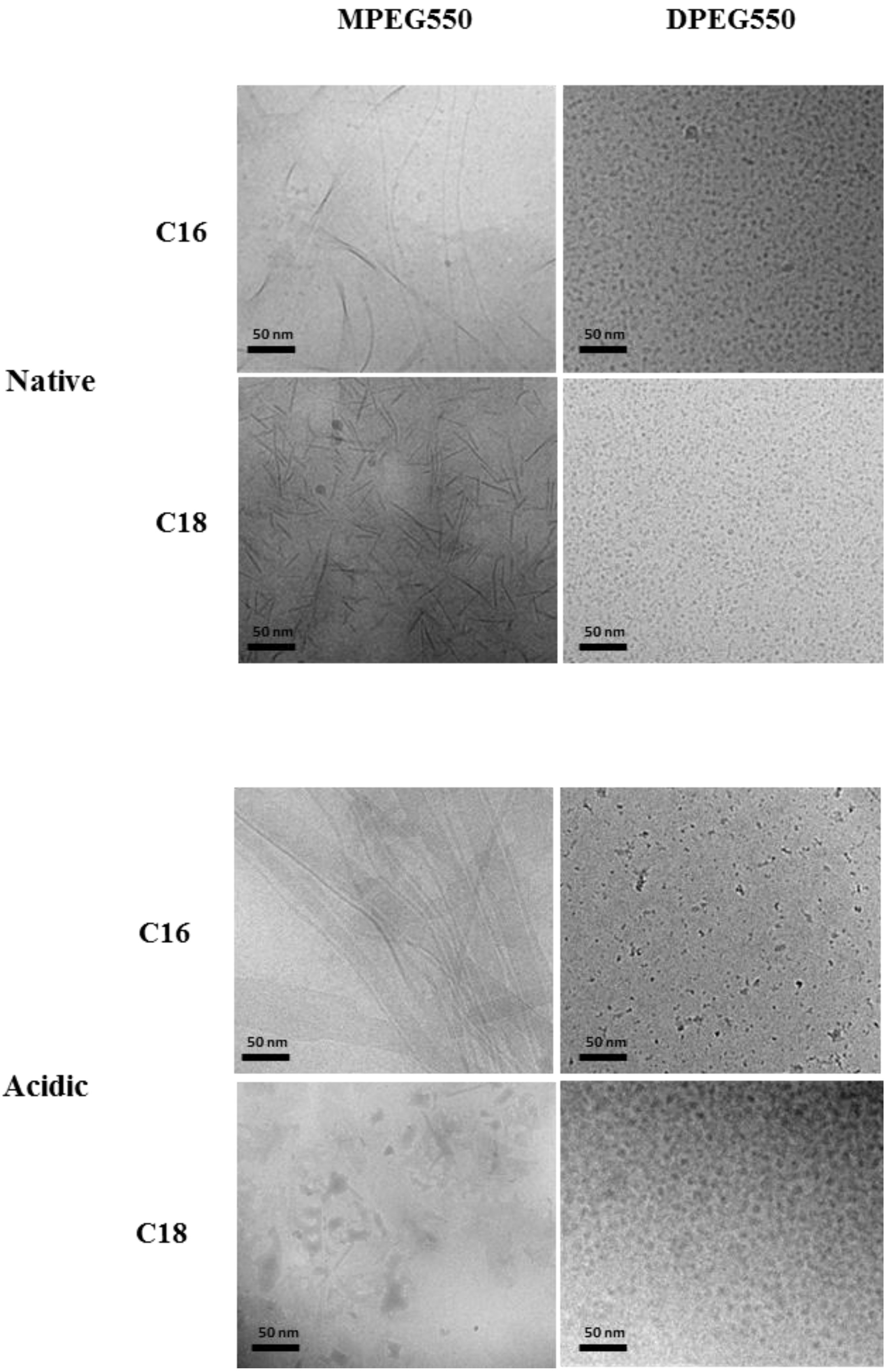

Figure S21: Cryo-TEM images comparing morphologies at native pH 7 and acidic pH 2 for 1 wt% solutions of the samples indicated.

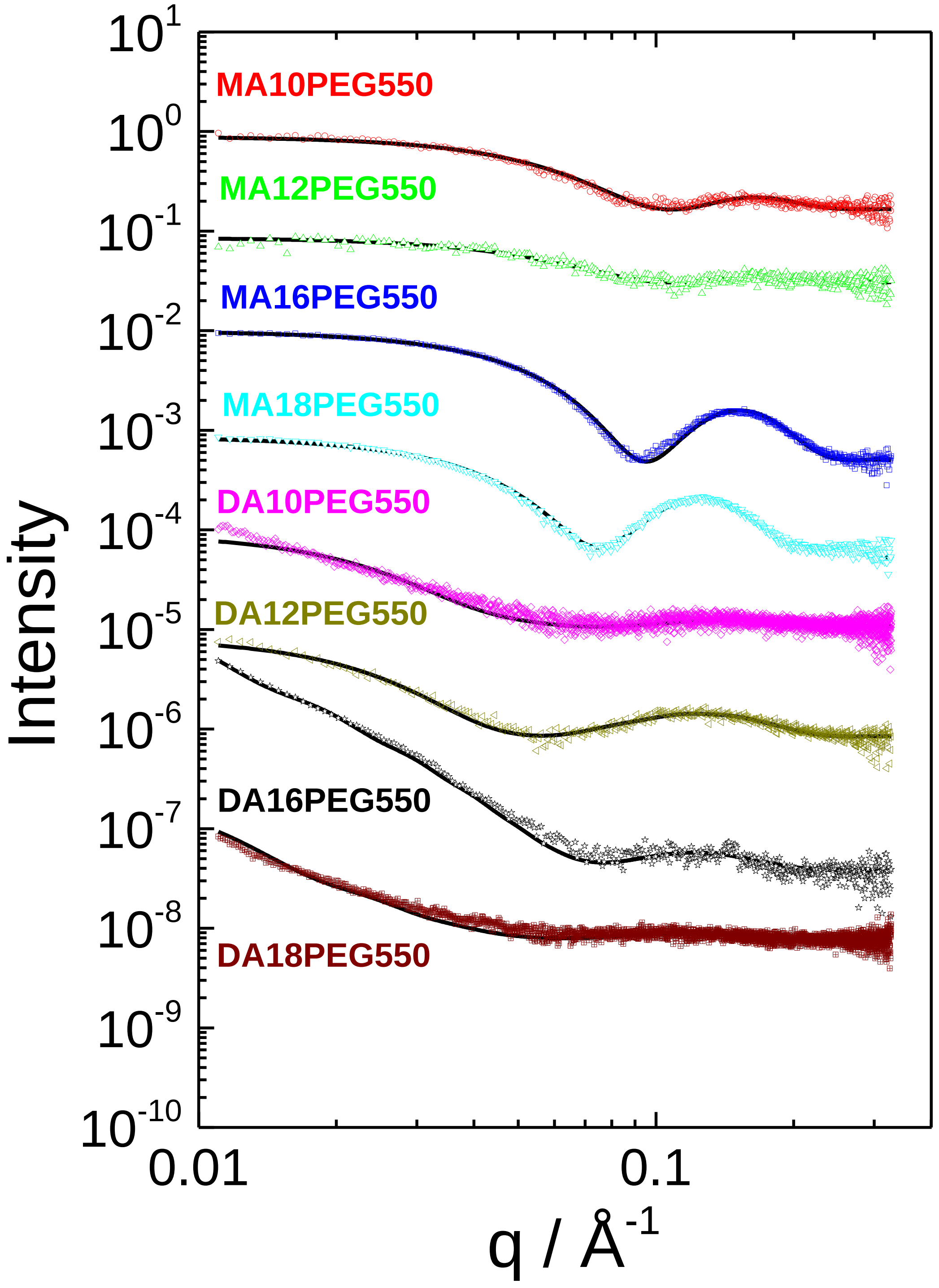

Figure S22: Measured SAXS data at pH 2 (open symbols) and fitted form factors (models and fitted parameters listed in SI Table S2). Data has been scaled to enable visualization, and only every 5th measured data point is shown.

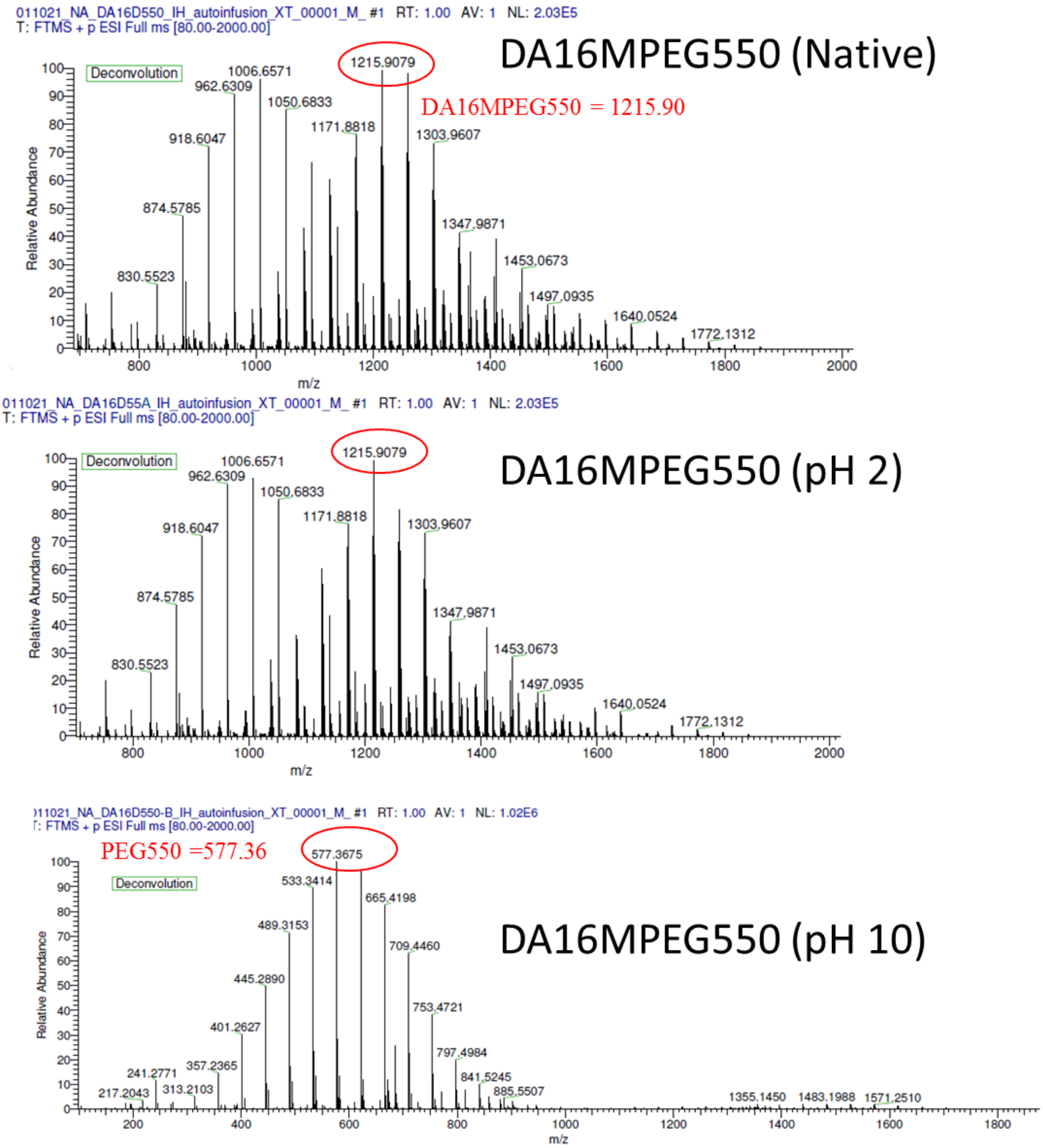

Figure S23: ESI-MS data for DA16MPEG550 at different pH values. The distribution of peaks reflects the distribution of PEG chain lengths in the commercial (Sigma-Aldrich polyethylene glycol methyl ether) sample.

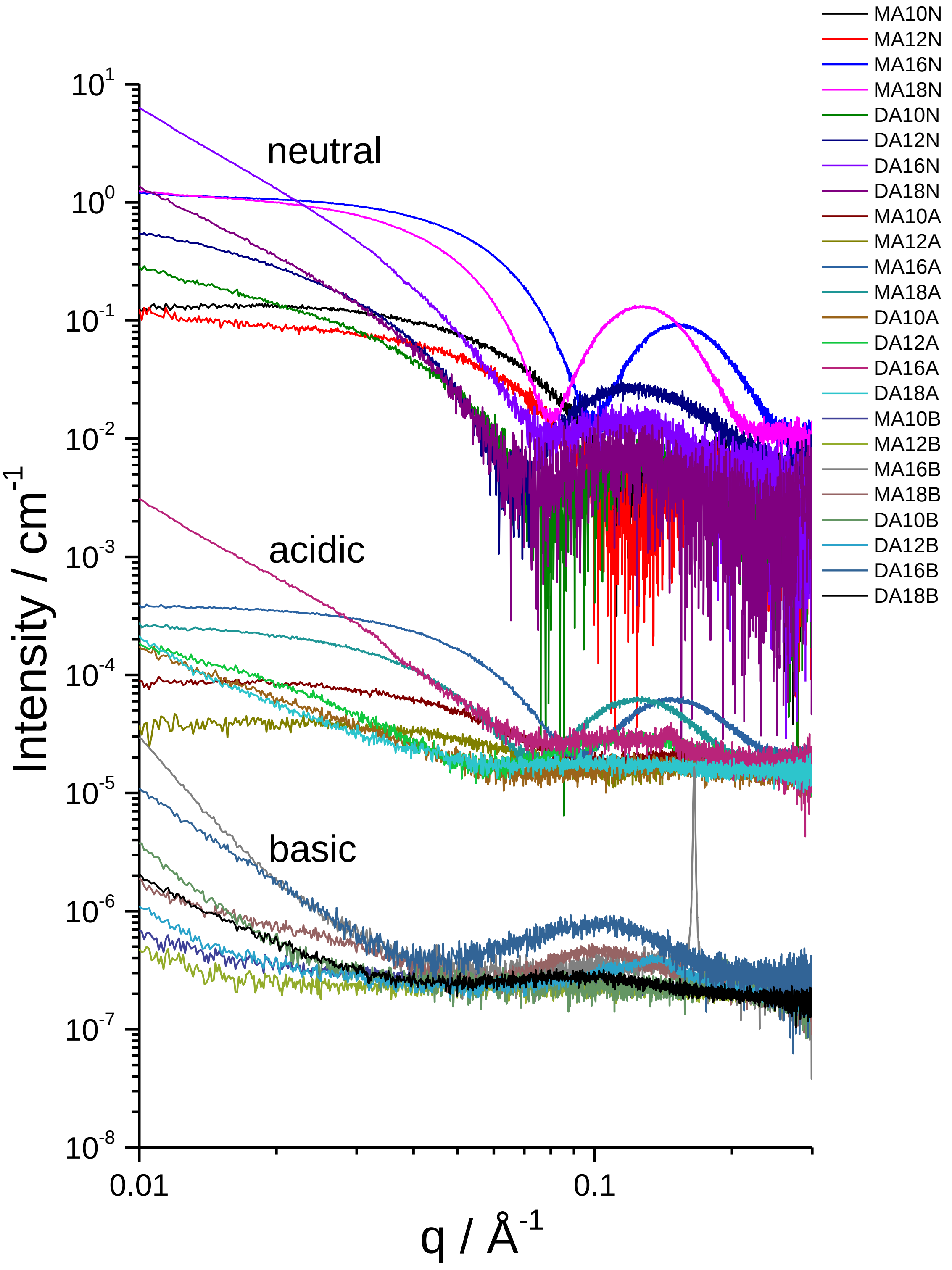

Figure S24: Comparison of SAXS data at neutral pH 7, acidic pH 2 and basic pH 10 for 1 wt% solutions of all samples as indicated.

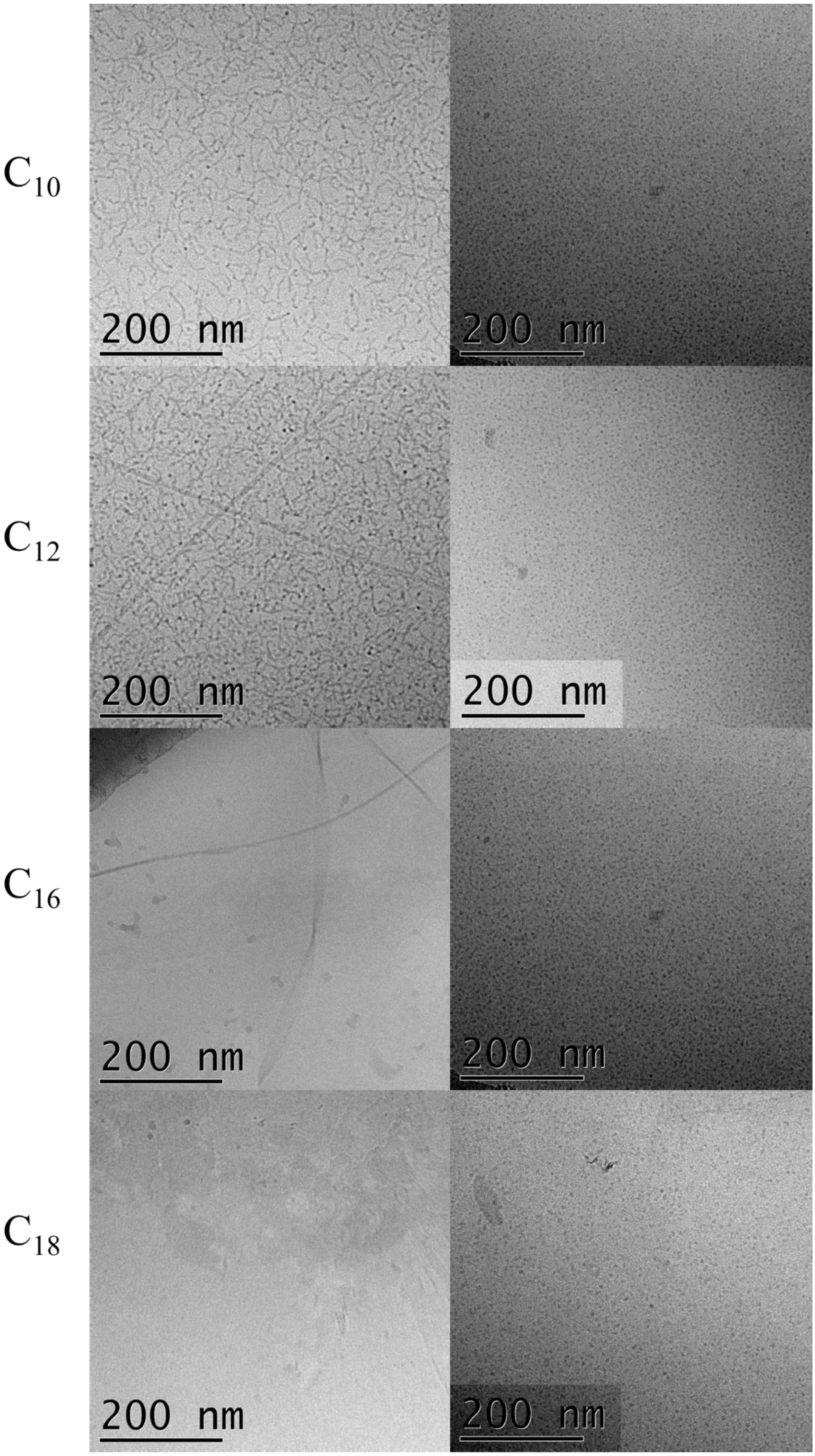

Figure S25: Cryo-TEM images for (left) DAmMPEG550 series and (right) the MAmDPEG550 series (all 1 wt% PBS buffer solutions).

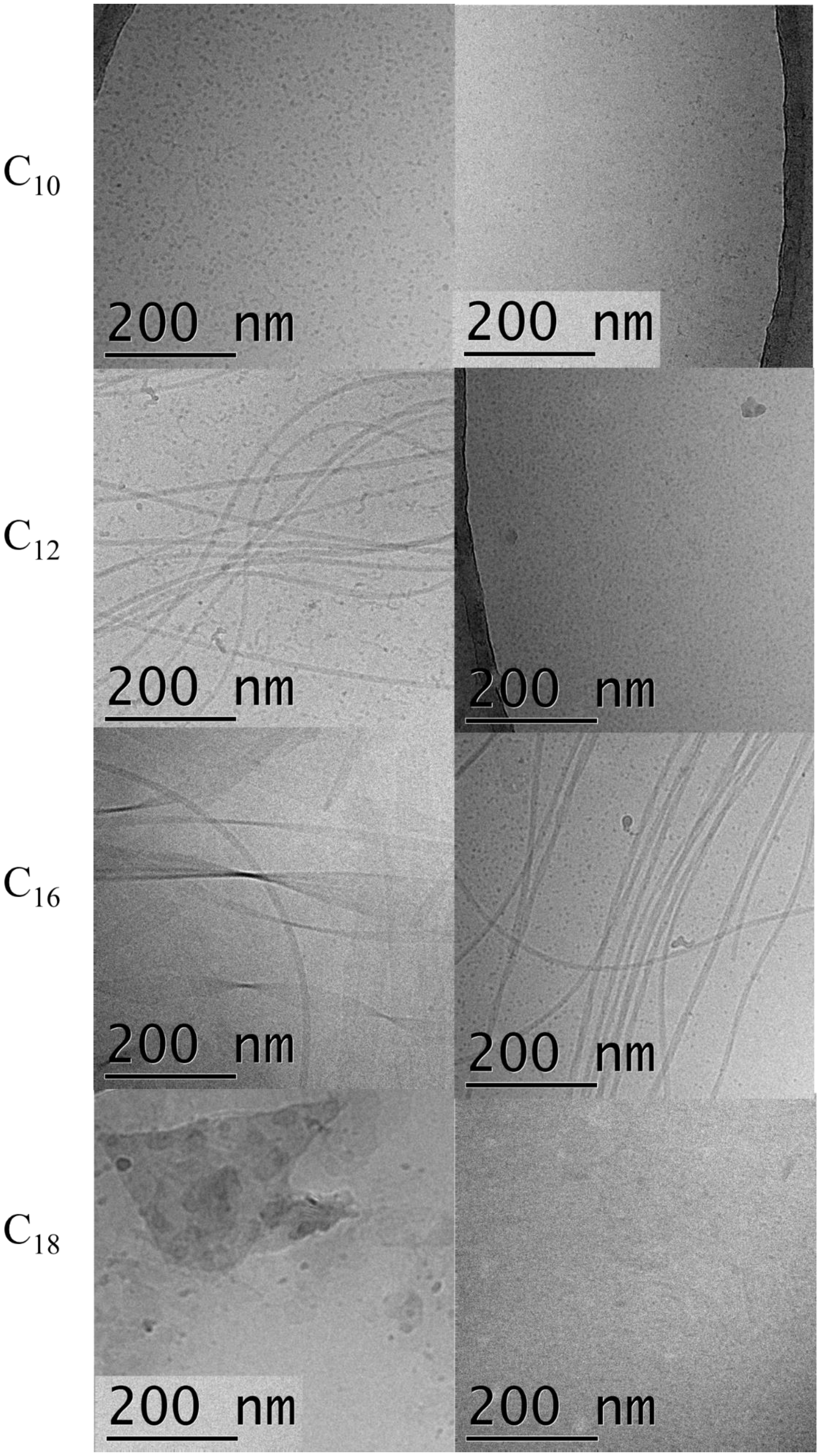

Figure S26: Cryo-TEM images for (left) DAmMPEG550 series and (right) the MAmDPEG550 series (all 1 wt% DMEM media solutions).

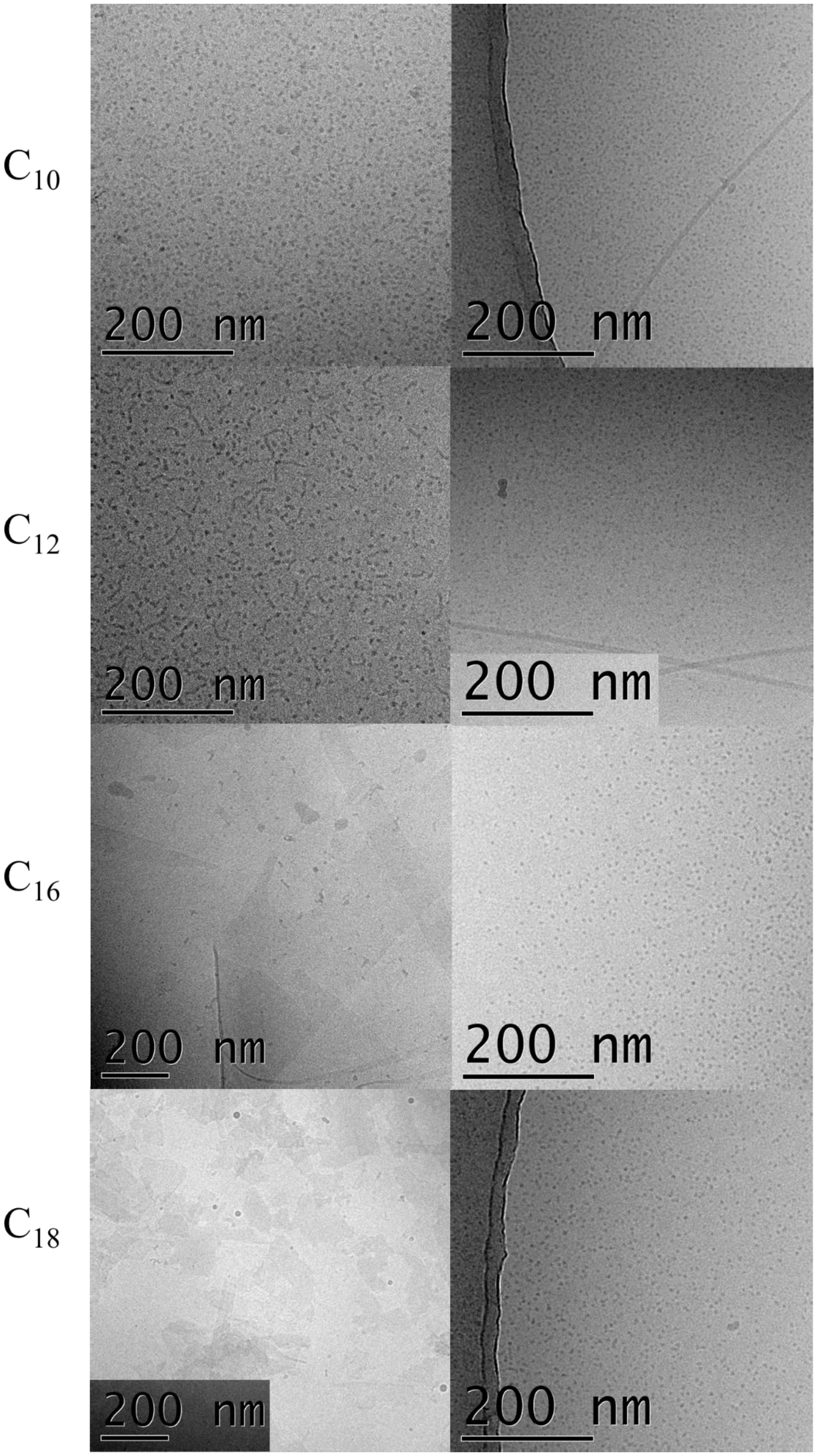

Figure S27: Cryo-TEM images for (left) DAmMPEG550 series and (right) the MAmDPEG550 series (all 1 wt% RPMI-1640 media solutions).

Table S1. Parameters extracted from the fitting of the SAXS data[a] at neutral pH. All samples were 1 wt% aqueous solutions.

	MA10-PEG550	MA12-PEG550	MA16-PEG550	MA18-PEG550	DA10-PEG550	DA12-PEG550	DA16-PEG550	DA18-PEG550[b]
$R_c \pm \Delta R_c$ [Å]	9.1 ± 0.7	10.1 ± 0.03	11.2 ± 0.8	12.2 ± 1.6				
R_s [Å]	34.0	34.0	36.4	43.7				
η_s [$cm^{-1/2}$]	7.8×10^{-7}	6.7×10^{-7}	2.1×10^{-6}	1.3×10^{-6}				
μ	-3.3	-4.1	-5.0	-8.7				
$t \pm \Delta t$ [Å]					27.1 ± 0.8	36.0 ± 3.0	40.0 ± 8.0	45.1 ± 1.0
η_{out} [cm^{-1}]					4.0×10^{-7}	5.5×10^{-7}	1.9×10^{-7}	1.8×10^{-7}
σ_{out} [Å]					8.0	6.0	4.4	1.8
η_{in} [cm^{-1}]					-7.0×10^{-7}	-1.1×10^{-6}	-3.6×10^{-8}	-6.4×10^{-8}
σ_{in} [Å]					2.8	2.8	2.0	1.6
D [Å]					130	150	500	500
C	4.0×10^{-4}	2.1×10^{-4}	1.1×10^{-3}	4.0×10^{-4}	3.0×10^{-4}	5.0×10^{-4}	3.9×10^{-4}	1.6×10^{-3}

[a] Data fitted using form factor of a Spherical Shell (model i) (all MA series samples) or Gaussian bilayer (all DA series samples) using the software SASfit.[2]

[b] Noisy data with low confidence on fit parameters

Key: Spherical Shell: core radius, R_c (Gaussian polydispersity ΔR_c), outer radius (including shell), R_s, scattering contrast of shell η_s, and core $\eta_c = \mu\eta_s$.. **Gaussian bilayer:** layer thickness t (Gaussian polydispersity Δt), scattering contrast of outer layers η_{out}, and inner layer η_{in}, Gaussian widths σ_{in} and σ_{out} of inner and outer layers respectively, D diameter (width) of layer system. **Background:** constant background, C.

Table S2. Parameters extracted from the fitting of the SAXS data[a] at pH = 2. All samples were 1 wt% aqueous solutions.

	MA10-PEG550	MA12-PEG550	MA16-PEG550	MA18-PEG550	DA10-PEG550	DA12-PEG550	DA16-PEG550	DA18-PEG550
$R_c \pm \Delta R_c$ [Å][b]	9.1 ± 0.7	10.1 ± 0.03	11.2 ± 0.8	12.2 ± 1.6				
R_s [Å][b]	34.0	34.0	36.4	43.7				
η_s [cm$^{-1/2}$]	6.3×10^{-7}	3.9×10^{-7}	1.3×10^{-6}	6.2×10^{-7}				
μ	-6.7	-5.6	-7.2	-11.8				
$t \pm \Delta t$ [Å] [b]					27.1 ± 0.8	36.0 ± 3.0	40.0 ± 8.0	45.1 ± 1.0
η_{out} [cm^{-1}]					2.4×10^{-7}	6.4×10^{-7}	1.9×10^{-7} [b]	1.8×10^{-7} [b]
σ_{out} [Å]					6.7	4.7	4.5	1.8
η_{in} [cm^{-1}]					-7.7×10^{-7}	-3.6×10^{-7}	-3.6×10^{-8} [b]	-6.4×10^{-8} [b]
σ_{in} [Å]					2.0	9.8	2.0	1.6
D [Å]					179	159	603	460
C	1.6×10^{-3}	1.5×10^{-3}	1.1×10^{-3}	1.5×10^{-3}	1.4×10^{-3}	1.7×10^{-3}	3.9×10^{-4}	1.6×10^{-3}

[a] Data fitted using form factor of a Spherical Shell (model i) (all MA series samples) or Gaussian bilayer (all DA series samples) using the software SASfit.[2]

[b] Parameter fixed at values in Table S1.

Noisy data with low confidence on fit parameters

Key: Spherical Shell: core radius, R_c (Gaussian polydispersity ΔR_c), outer radius (including shell), R_s, scattering contrast of shell η_s, and core $\eta_c = \mu\eta_s$.. **Gaussian bilayer:** layer thickness t (Gaussian polydispersity Δt), scattering contrast of outer layers η_{out}, and inner layer η_{in}, Gaussian widths σ_{in} and σ_{out} of inner and outer layers respectively, D diameter (width) of layer system. **Background:** constant background, C.

(1) Cowieson, N. P.; Edwards-Gayle, C. J. C.; Inoue, K.; Khunti, N. S.; Doutch, J.; Williams, E.; Daniels, S.; Preece, G.; Krumpa, N. A.; Sutter, J. P.; Tully, M. D.; Terrill, N. J.; Rambo, R. P., Beamline B21: high-throughput small-angle X-ray scattering at Diamond Light Source. *J. Synchrotron Rad.* **2020,** *27*, 1438-1446.

(2) Bressler, I.; Kohlbrecher, J.; Thünemann, A. F., SASfit: a tool for small-angle scattering data analysis using a library of analytical expressions. *J. Appl. Cryst.* **2015,** *48*, 1587-1598.
